# Excessive endometrial PlGF- Rac1 signalling underlies endometrial cell stiffness linked to pre-eclampsia

**DOI:** 10.1038/s42003-024-06220-7

**Published:** 2024-05-04

**Authors:** Janet P. Raja Xavier, Carmela Rianna, Emily Hellwich, Iliana Nikolou, Aditya Kumar Lankapalli, Sara Y. Brucker, Yogesh Singh, Florian Lang, Tilman E. Schäffer, Madhuri S. Salker

**Affiliations:** 1 Department of Women’s Health, University of Tübingen, Tübingen, Germany; 2 Institute of Applied Physics, University of Tübingen, Tübingen, Germany; 3 Department of Biology, Ineos Oxford Institute of Antimicrobial Research, Oxford, UK; 4 Institute of Medical Genetics and Applied Genomics, University of Tübingen, Tübingen, Germany; 5 Department of Physiology, University of Tübingen, Tübingen, Germany

**Keywords:** Molecular medicine, Reproductive disorders

## Abstract

Cell stiffness is regulated by dynamic interaction between ras-related C3 botulinum toxin substrate 1 (Rac1) and p21 protein-activated kinase 1 (PAK1) proteins, besides other biochemical and molecular regulators. In this study, we investigated how the Placental Growth Factor (PlGF) changes endometrial mechanics by modifying the actin cytoskeleton at the maternal interface. We explored the global effects of PlGF in endometrial stromal cells (EnSCs) using the concerted approach of proteomics, atomic force microscopy (AFM), and electrical impedance spectroscopy (EIS). Proteomic analysis shows PlGF upregulated RhoGTPases activating proteins and extracellular matrix organization-associated proteins in EnSCs. Rac1 and PAK1 transcript levels, activity, and actin polymerization were significantly increased with PlGF treatment. AFM further revealed an increase in cell stiffness with PlGF treatment. The additive effect of PlGF on actin polymerization was suppressed with siRNA-mediated inhibition of Rac1, PAK1, and WAVE2. Interestingly, the increase in cell stiffness by PlGF treatment was pharmacologically reversed with pravastatin, resulting in improved trophoblast cell invasion. Taken together, aberrant PlGF levels in the endometrium can contribute to an altered pre-pregnancy maternal microenvironment and offer a unifying explanation for the pathological changes observed in conditions such as pre-eclampsia (PE).

## Abstract (web summary)

High levels of placental growth factor can result in a stiffer microenvironment milieu impeding trophoblast invasion of endometrial cells prior to pregnancy, providing insight into the pathogenesis of pre-eclampsia.

## Introduction

Pregnancy is dependent on the transformation of the endometrium, a process driven by the differentiation of endometrial stromal cells (EnSCs) into specialized decidual cells^1^. The decidua contributes to early nutrient exchange, production of cytokines, and growth factors as well as supports the development of new blood vessels, modulates extravillous trophoblast (EVT) invasion, and acts as a protective barrier against infections and maternal host immune responses^2^. Disturbances in the uterine microenvironment are associated with miscarriages and third-trimester perinatal morbidity and mortality caused by pre-eclampsia (PE) and intrauterine growth restriction, small-for-gestational age, pre-term birth, and stillbirth^3–5^. It is increasingly evident that abnormal levels of inflammatory and growth/signaling factors produced in the decidua during early pregnancy play a decisive role in the deregulation of the local microenvironment leading to several disorders of pregnancy^6,7^. The endometrial contribution to the etiology of PE remains elusive. A recent study, albeit at the transcriptomic level, highlighted the role of poor decidualisation in PE^8^. However, the exact molecular mechanism of how a defective endometrial microenvironment in pre-pregnancy can lead to pregnancies complicated by PE remains unclear.

Placental Growth Factor (PlGF) was first identified in the placenta and is up-regulated in several pathological conditions such as tumor malignancy, sepsis, arthritis, and diabetic retinopathy^9–12^. PlGF primarily exerts its action on endothelial cells^13^, but a plethora of studies now support the role of PlGF in non-endothelial tissues including the liver, skeleton, and thyroid^14–17^. PlGF is present in the human endometrium, exhibiting temporal changes across the menstrual cycle and reinforces uterine angiogenesis^15^. Expression of PlGF has been reported in the decidua, placenta, uterine natural killer cells, and trophoblasts cells^15^. An intricate balance between its angiogenic and inflammatory roles in the endometrium is considered important for successful conception^14^. Several studies show up-regulation of endometrial PlGF causes switching of the controlled uterine angiogenesis to a severe inflammation cascade leading to tissue damage and early pregnancy losses^14^. It was further shown that PlGF modifies cellular motility and adhesion with important roles in early pregnancy^15^. Low levels of PlGF are associated with PE and used as a clinical diagnostic marker for PE prediction^13,18^, but it remains uncertain whether dysregulated low PlGF level is a cause or consequence of PE progression.

Ras-related C3 botulinum toxin substrate 1 (Rac1) is a major player of the Rho family of small GTPases that controls multiple cell signalling pathways such as cytoskeleton organization, cell proliferation, apoptosis and immune activation^19^. The major downstream cellular target of activated Rac1 is p21(Rac1)-activated kinase (PAK1)^20^ and activation of this pathway reverses the process of polymerization brought on by ARP2/3 activity, converting F-actin filaments into G-actin monomers^21^. The actin cytoskeleton provides the structural scaffold for a cell and principally determines its mechanical properties. Consequently, any alteration of actin polymerization is anticipated to modify cell stiffness^22,23^. Regulatory proteins of the actin cytoskeleton including Rac1 play a pivotal role during implantation^24^. Therefore, the ability of endometrial cells to support the ongoing pregnancy requires a delicate balance of reorganization of the actin cytoskeleton to promote EVT invasion and to restrict over invasion^25,26^. Interestingly, PlGF plays a pivotal role in actin regulation and cytoskeletal rearrangement in breast cancer and leukemic cells making them stiffer^16,27^. Furthermore, PE is associated with impaired cytoskeleton rearrangements and increased arterial stiffness^28,29^. However, a functional mechanism has not been directly investigated. During early pregnancy, EVTs invade the endometrium cluster around the spiral arteries and induce their remodelling^30^. Differences in stiffness within the endometrium could influence EVT migration. Given that PE is characterized by shallow EVT invasion, it is tempting to speculate that a stiffer endometrium prior to pregnancy may indeed contribute to the pathogenesis of PE in part by hindering invasion.

Taken together, here we postulate that excessive PlGF levels in the endometrium contribute to impaired cytoskeletal rearrangements and stiffness and thus participate in the pathophysiology of PE. To test this hypothesis, manual mining of RNA sequencing (RNA-seq) data shows that at a pathway level, several biological processes including extracellular structure, actin, and cell-matrix organization were affected in the decidua prior to the onset of PE. We, therefore, sought to determine whether high PlGF affects the actin turnover and cell stiffness of EnSCs. We report that high PlGF (20 ng/ml for 6 days) leads to dynamic changes in the mechanical cellular properties in EnSCs. We provide evidence that PlGF increases Rac1 activity, PAK1 phosphorylation, actin polymerization, and cell stiffness. Lastly, we show that PlGF-induced Rac1 and actin dysregulation can be improved with the pharmacological substance pravastatin. Interestingly, improved endometrial actin dynamics with pravastatin also enhanced trophoblast (BeWo) cell invasion through endometrial cell monolayers.

## Results

### PlGF induces proteome changes, pointing to the regulators of cellular actin machinery and ECM organization

To verify the expression kinetics of PlGF across the menstrual cycle, we performed *insilico* analysis (*GEO2052*). Whole genome molecular phenotyping of human endometrial tissue enabled the identification of PlGF molecular signature across the menstrual cycle in normo-ovulatory women. We observe that PlGF is higher in the proliferative phase (n = 4) with expression levels declining across the secretory phase (n = 6) of the cycle (Fig S1a). Next, we explored whether PE pathophysiology is associated with dysregulated levels of endometrial PlGF and cytoskeleton-activating Rho-GTPases proteins like PAK1. We verified their expression levels obtained from microarray and gene expression studies in uteroplacental units from 10 preeclamptic women and 10 women with healthy pregnancies. We found that PlGF and PAK1 transcripts were upregulated in term decidua’s (Fig S1b) of preeclamptic women (*GEO2548*) compared to the decidua from healthy pregnancies.

Furthermore, we mined a publicly available RNA sequencing array (in silico analysis, *GSE172381*) from endometrial biopsies obtained from women who developed PE in a previous pregnancy (n = 24) and controls who never had PE (*n* = 16). We verified transcriptomic alterations in the endometrium from patients with a history of PE. Gene Ontology analysis of the gene signature identified six classes and 43 enriched biological processes (FDR < 0.05). These pathways were mainly associated with cell signalling, cell motility, cytoskeleton extracellular matrix, and reproductive process (Fig S1c). These pathways are hallmarks of impaired endometrial function and PE pathogenesis. We identified 93 genes representative of the altered pathways in PE participating in the extracellular matrix organization, cell motility, and in cytoskeleton (Fig S1d). These *insilico* findings discussed above provided us with a positive cue for a possible correlation between dysregulated decidual PlGF and altered endometrial mechanics as observed in PE pathogenesis.

**Fig. 1:**
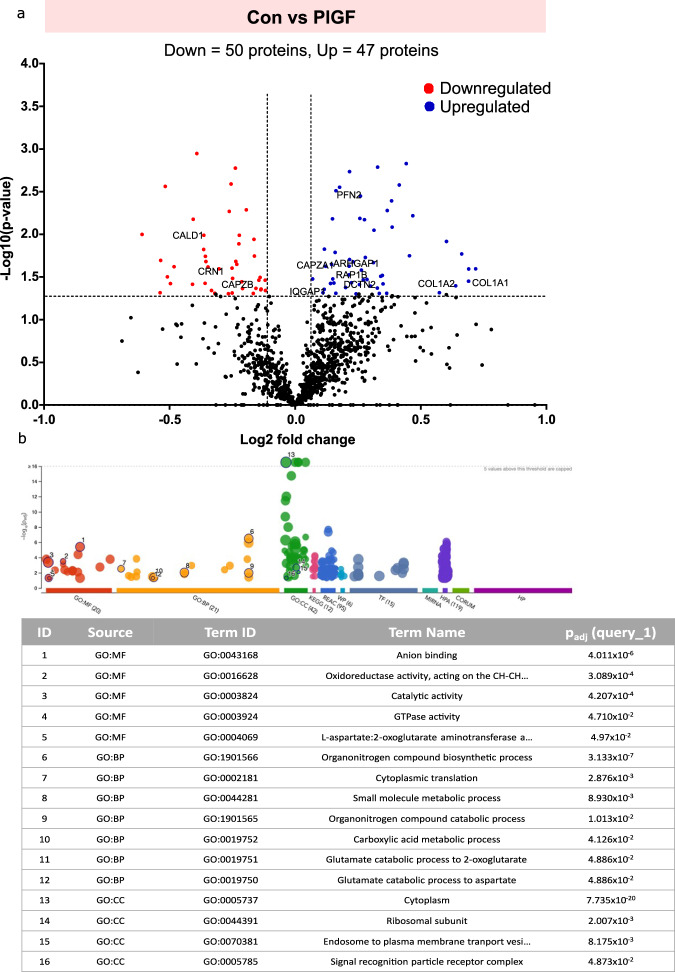
Protein expression profile of EnSCs cell lysates treated with PlGF. **a** Volcano plot showing upregulated (blue) and downregulated (red) proteins in PlGF-treated EnSCs (*n* = 3) compared to untreated EnSCs (*n* = 3) (control). Each point represents one protein; black points are the rest of the proteins obtained in the global proteomic analysis. The significance threshold range is 0.05 and the fold change threshold is −1 and +1. **b** Gene Ontology analysis of the protein signature associated with PlGF treatment in EnSCs shows enriched biological processes.

To identify a putative link between endometrial cells and cell stiffness, EnSCs were treated with PlGF (20 ng/ml for 6 days). The cells were then harvested for proteomics. Proteomic analysis was performed by comparing protein expression patterns in control (*n* = 3) and PlGF (*n* = 3) treated EnSCs. This comparison revealed a total of 97 dysregulated proteins. Differentially regulated proteins were shown in volcano plots as seen in Fig. 1a. A total of 47 upregulated (blue) and 50 (red) downregulated differentially regulated proteins were identified as being associated with PlGF treatment in EnSCs. Upregulated proteins include those involved in actin cytoskeleton regulation, such as DCTN2, PFN2, RAP1B, and CAPZA1; and proteins associated with GTPase regulation (IQGAP1 and ARHGAP1), and extracellular matrix organization proteins (COL1A2, COL1A1, and TUBA1A). Some of the downregulated proteins include actin-stabilizing proteins such as CAPZB, CNN1, and CALD1. Gene Ontology analysis of the protein signature associated with PlGF treatment in EnSCs also identified biological process associated with GTPases activity (Fig. 1b). Further to validate the above finding, we studied the effect of PlGF on ECM markers of EnSCs with qPCR. We show that PlGF enhanced gene expression of ECM-associated markers (*ACTA2, COL1A1, COL1A2, COL3A1, COL4A1*) and matrix metalloprotease markers (*MMP2 and MMP9*) in EnSCs confirming the increased ECM associated transcriptome changes as seen in proteomics data (Fig S2a, b). These findings suggested that PlGF exerts a pro-stiffness phenotype with actin regulation and GTPases activation signaling pathway being one of the most common GO terms.

### PlGF upregulates cytoskeletal regulators Rac1 and PAK1 in endometrial stromal cells

**Fig. 2:**
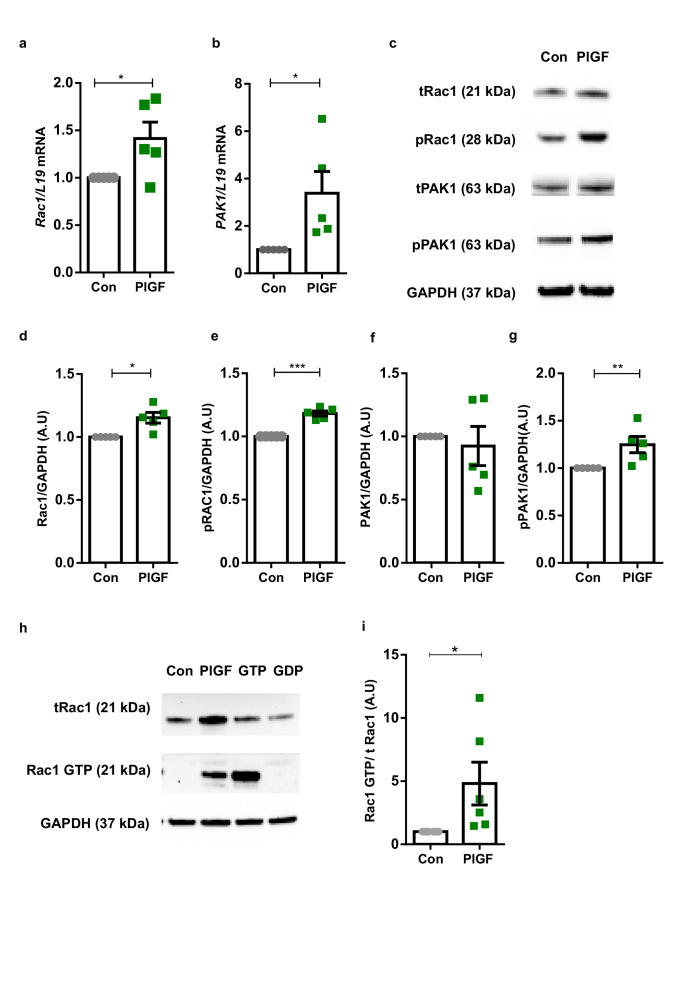
Effect PlGF on transcript and protein levels of Rac1 and PAK1 cytoskeletal regulators. **a** Arithmetic mean ± SEM of Rac1 transcript level in EnSCs on treatment with PlGF (*n* = 5, *p < 0.05). **b** Arithmetic mean ± SEM of PAK1 transcripts levels in EnSCs on treatment with PlGF (*n* = 5, *p < 0.05). **c** Original western blots representing total and phosphorylated levels of Rac1 and PAK1 targets with GAPDH used as a loading control. **d** Arithmetic mean ± SEM of protein levels of total Rac1 in untreated and PlGF-treated EnSCs (*n* = 5, *p < 0.05,). **e** Arithmetic mean ± SEM of protein levels of phosphorylated Rac1in untreated and PlGF-treated EnSCs (*n* = 5, ***p < 0.01). **f** Arithmetic mean ± SEM of protein levels of total PAK1 in untreated and PlGF-treated EnSCs. **g** Arithmetic mean ± SEM of protein levels of phosphorylated PAK1 in untreated and PlGF-treated EnSCs (*n* = 5, **p < 0.01). **h** Original western blots of total Rac1, Rac1 GTP, and GAPDH from Rac1 Pull down assay. **i** Arithmetic mean ± SEM of protein levels of activated form of Rac1, as Rac1GTP normalised to total Rac1(*n* = 6, *p < 0.05) in EnSCs on treatment with PlGF and untreated controls. All the above data represented here is normalized to control samples. The statistical significance was tested with an Unpaired *t*-test with Welch’s correction.

Next, we investigated to gain insights into the effect of PlGF on small G protein or GTPase Rac1-dependent regulation of actin organization and polymerization in endometrial stromal cells. Rac1-PAK1 cellular signaling pathway accounts for a pivotal role in the reorganization of the actin filaments, hence the effect of PlGF on Rac1 expression and activity was verified. PlGF treatment (20 ng/ml for 6 days) was followed by a significant increase of both Rac1 (*p < 0.05) and PAK1 (*p* < 0.05) transcript levels normalized to L19 transcript levels (Fig. 2a, b). Moreover, PlGF treatment was also followed by a significant increase in total (**p < 0.01) and phosphor-Rac1 (***p < 0.001) protein levels (Fig. 2c–e and Supplementary information).

Rac1 activation is known to trigger phosphorylation of PAK1, which in turn regulates the actin turnover dynamics. As shown in Fig. 2c –g and Supplementary information treatment with PlGF for 6 days increased PAK1 total protein level and phosphorylated levels (*p < 0.05), pointing to an increase of PAK1 activity in endometrial cells. Further, to validate the activation of Rac1 protein, Rac1 activation assay (immunoprecipitation) was employed. Rac1 activity was assayed by determining the amount of GTP-bound (activated) Rac1 that was precipitated by a GST-PAK1 fusion protein. As evident in Fig. 2h, i and Supplementary information, PlGF treatment dramatically increased the activity of Rac1(GTP) (*p < 0.05) compared to control. Actin regulator Rac1 acts as a key molecule in regulating cell migration^31^. Thereby, we studied the migratory potential of PlGF-treated EnSCs using a wound healing assay. PlGF treatment enhanced cell migration in EnSCs (Fig S3a, b) without a change in cell proliferation (Fig S3c, d) as verified by a BrdU proliferation ELISA and MTS.

### PlGF downregulates actin depolymerization and augments cell stiffness in endometrial stromal cells

**Fig. 3:**
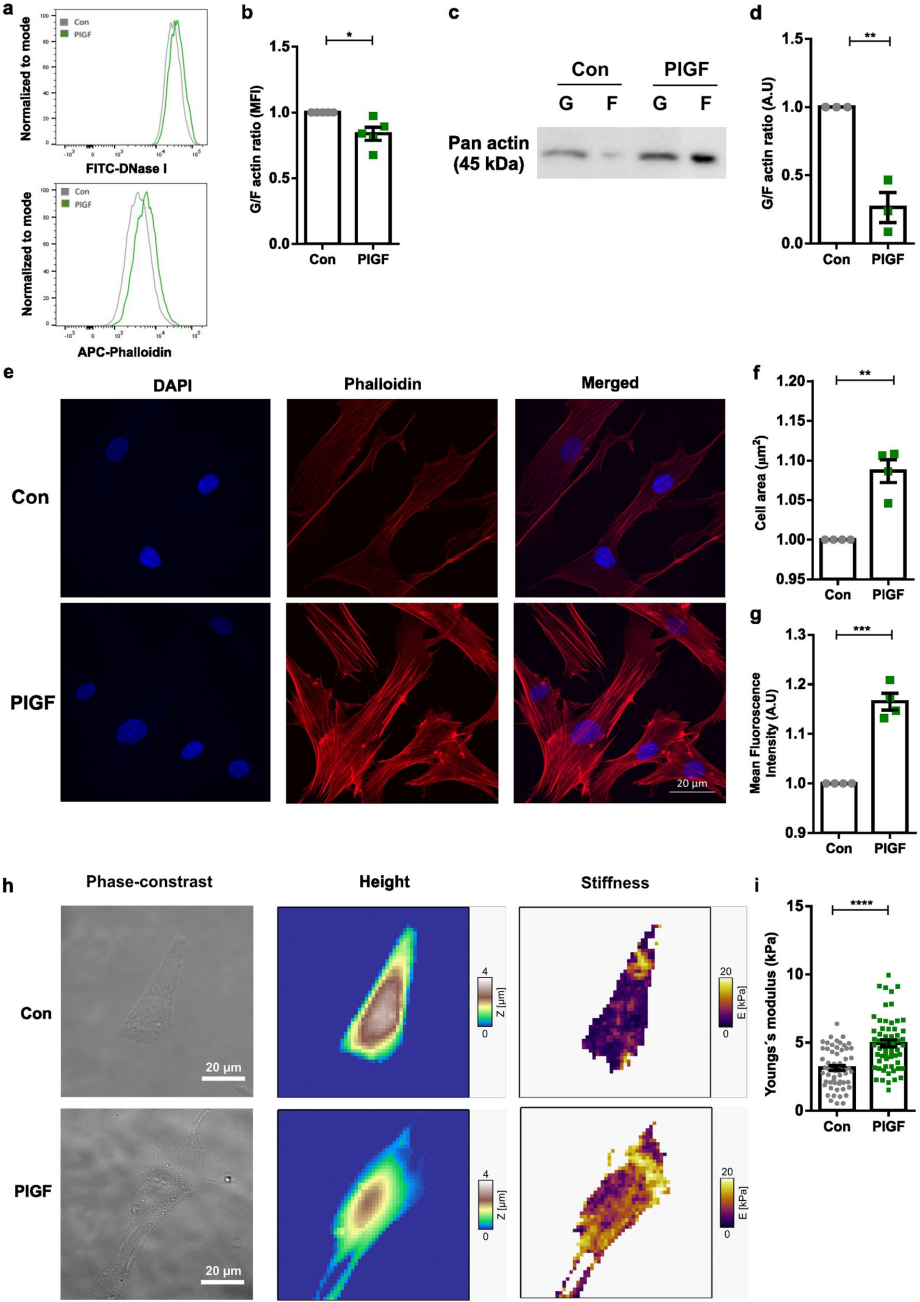
Effect of PlGF on actin polymerization and cell stiffness in EnSCs. **a** Representative original histogram of DNaseI (G-actin; Left) and Phalloidin (F-actin; Right) binding in EnSCs after 6 days of treatment without and with PlGF. **b** Arithmetic means ± SEM of mean fluorescence intensity (MFI) of G-actin over F-actin ratio in EnSCs after 6 days of treatment without and with PlGF (*n* = 5, *p < 0.05). **c** Representative original western blot of soluble G-actin and filamentous F-actin in human endometrial EnSCs after 6 days of treatment with PlGF. **d** Arithmetic mean ± SEM of G-actin over F-actin ratio in EnSCs after 6 days treatment with PlGF (*n* = 3, **p < 0.01). **e** Original immunofluorescence images of eflour650-phalloidin binding to F-actin (red) and DAPI staining for cell nuclei (blue) in EnSCs treated with or without PlGF. **f** Arithmetic mean ± SEM individual cells were measured) of cell area (µm^2^) measured in EnSCs post treatment with PlGF (*n* = 4, **p < 0.01). **g** Arithmetic mean ± SEM of mean fluorescence intensity (MFI) measured from immunofluorescence images in EnSCs post-treatment with PlGF (*n* = 4, ***p < 0.001). All the above data represented here are normalized to control cells. The statistical significance was tested with an Unpaired *t*-test with Welch’s correction. **h** AFM analysis of cell stiffness and cell morphology in untreated EnSCs and cells treated with PlGF for 6 days at 20 ng/ml. Representative optical phase contrast images, height images, and AFM stiffness images measuring the Young’s modulus. **i** Arithmetic mean ± SEM of Young’s modulus (cell stiffness) (*n* = 60, ****p < 0.0001). The statistical significance was tested with a non-parametric Mann-Whitney U test.

Activation of Rac1 in cells decreases the G/F actin ratio, which in turn enhances cellular stiffness. To test this, we next investigated whether the marked alterations of cell stiffness following PlGF treatment in stromal cells were marked by respective alterations with actin polymerization dynamics. Interestingly, as evident from both flow cytometry (Fig. 3a, b and S4, *p < 0.05) and western blotting analysis (Fig. 3c, d and Supplementary information, **p < 0.01), a 6-day treatment of EnSCs with PlGF significantly decreased the ratio of soluble G-actin over filamentous F-actin, reflecting the polymerization of actin filaments. Fluorescent images of F-actin organization (Fig. 3e and S5, red) and its concomitant changes under PlGF treatment show a profound reorganization of the actin cytoskeleton with an increased cell area (Fig. 3f, **p < 0.01) and enhanced fluorescence intensity (Fig. 3g, ***p < 0.001).

Filamentous actin (F-actin) is a structural cytoskeleton protein known to play an important role in maintaining cellular and tissue structure. To determine whether PlGF induces structural changes and modifies mechanical stiffness in EnSCs, atomic force microscopy (AFM) was performed on live EnSCs after treatment with PlGF for 6 days (20 ng/ml). Individual cells were subsequently imaged with AFM in the force mapping mode to quantify the cell stiffness (Fig. 3h). Comparison of the mean stiffness revealed that PlGF-treated EnSCs are significantly stiffer (〈*E*〉 = 4.94 kPa) than the control cells (〈*E*〉 = 3.13 kPa) (Fig. 3i, ****p < 0.0001). EGF, a known Rac1 activator, was used as a positive control for an increase in cell stiffness (Fig S6). Additionally, to infer the change in stiffness in a more relevant physiological environment, EnSCs were seeded onto PDMS substrates (bulk stiffness 1–2 kPa) and subjected to PlGF treatment as described above. We observed PlGF-treated EnSCs seeded on top of a soft substrate as PDMS gels, still showed an increase in cellular stiffness compared to untreated EnSCs (Fig S7).

EnSCs undergo a differentiation process termed decidualization, initiated by progesterone during the early secretory phase^3,32^. Decidualization of stromal cells is critical to regulate trophoblast invasion to mediate pregnancy establishment and progression^1,32^. Therefore, we evaluated the effect of aberrant PlGF on EnSCs during decidualization in vitro. Further, we verified the effect of PlGF on actin regulators and cell stiffness of decidualized EnSCs. Our results show that PlGF impaired the decidualization potential in EnSCs with a decrease in transcript levels of markers such as prolactin and insulin-like growth factor binding protein-1 (Fig S8). The reduced decidualization markers were accompanied by decrease in Rac1 activity (Fig S9a) and a decrease in G/F actin ratio (Fig S9b, c) with increased cell stiffness (Fig S10) in PlGF-treated decidualized EnSCs compared to decidualized EnSCs. These results confirm that aberrant PlGF levels in the endometrium would impede the decidualizing behaviour of EnSCs with negative regulation of decidual markers, actin dynamics and cell stiffness.

### PlGF influence on endometrial actin regulation is mediated by Rac1, PAK1 and WAVE2 signalling effectors

**Fig. 4:**
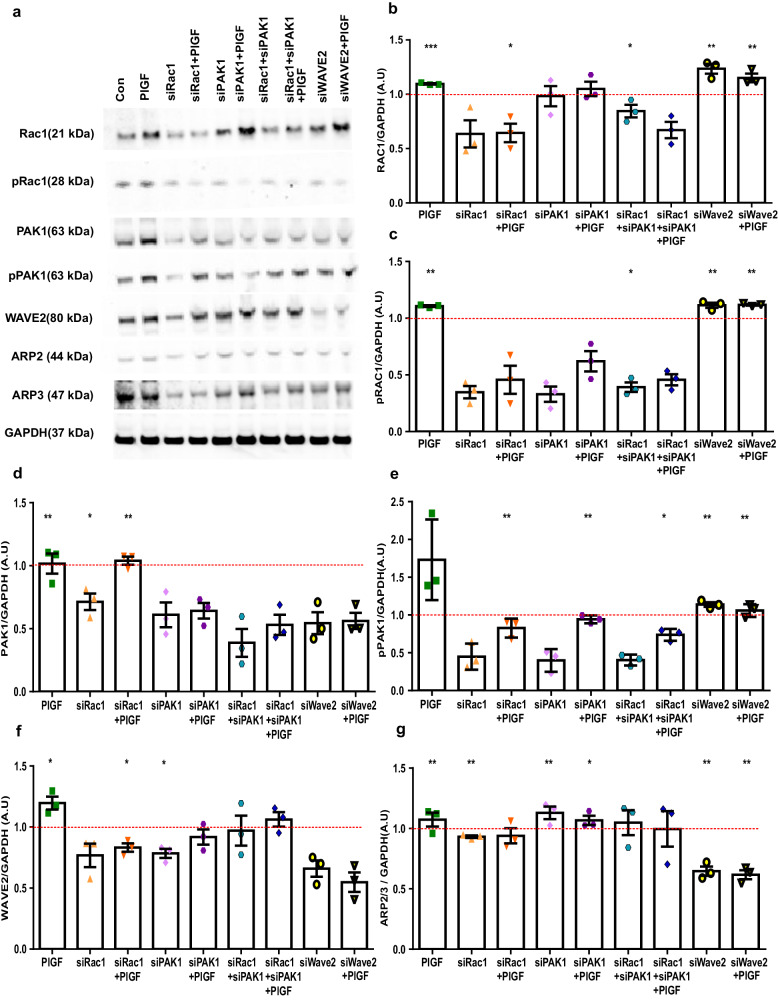
Effect of siRNA on PlGF-activated Rac1-PAK1 signaling cascade in EnSCs. **a** Original western blots of total and phosphorylated levels of Rac1, pRac1, PAK1and pPAK1, WAVE2, and ARP2/3 targets with GAPDH as loading control. **b** Arithmetic means ± SEM of protein levels of total Rac1 in siRNA Rac1/PAK1/WAVE2 or in combination with and without PlGF treatment (*n* = 3, *p < 0.05, **p < 0.01, ***p < 0.001). **c** Arithmetic means ± SEM of protein levels of phosphorylated Rac1 in siRNA Rac1/PAK1/WAVE2 or in combination with and without PlGF treatment (*n* = 3, *p < 0.05, **p < 0.01). **d** Arithmetic means ± SEM of protein levels of total PAK1 in siRNA Rac1/PAK1/WAVE2 or in combination with and without PlGF treatment (*n* = 3, *p < 0.05, **p < 0.01). **e** Arithmetic means ± SEM of protein levels of phosphorylated PAK1 in siRNA Rac1/PAK1/WAVE2 or in combination with and without PlGF treatment (*n* = 3, *p < 0.05, **p < 0.01). **f** Arithmetic means ± SEM of protein levels of WAVE2 in siRNA Rac1/PAK1/WAVE2 or in combination with and without PlGF treatment (*n* = 3, *p < 0.05). **g** Arithmetic means ± SEM of protein levels of ARP2 over ARP3 ratio in siRNA Rac1/PAK1/WAVE2 or in combination with and without PlGF treatment (*n* = 3, *p < 0.05, **p < 0.01). All the above data represented here is normalized to control cells. The control is represented as red dotted lines in the graphs. The statistical significance was tested between control and treated samples with an Unpaired t-test with Welch’s correction.

To prove unequivocally that PlGF directly modulates the Rac1-PAK1 signaling pathway, EnSCs were first treated with PlGF for 4 days followed by transfection with Rac1, PAK1, and/or WAVE2 siRNAs for 48 h, and then continued with PlGF treatment for 2 more days (20 ng/ml). Rac1 gene silencing was efficient (40-50% silencing) and co-treatment with PlGF further suppressed both total and phosphorylated Rac1 (50-60% silencing) and PAK1 protein expression in EnSCs (Fig. 4a–d and Supplementary information, *p < 0.05, **p < 0.01, ***p < 0.001). Similarly, inhibition of PAK1 with siPAK1 ( ± PlGF) again paralleled the effect of Rac1 gene silencing, significantly downregulating total (40-50% silencing) and phosphorylated levels of PAK1(50-60% silencing) protein activity with and without PlGF (Fig. 4a, d, e and Supplementary information, **p < 0.01).

Further, we investigated the influence of PlGF on Rac1 regulatory proteins responsible for actin nucleation and turnover such as WAVE2 and ARP2/3. We demonstrated that, PlGF significantly increased protein levels of WAVE2 and ARP2/3 ratio in EnSCs (Fig. 4f, g and Supplementary information, *p < 0.05, **p < 0.01). Further, silencing of Rac1 or WAVE2 with and without PlGF treatment, was also followed by a decrease in expression levels of these proteins validating their activation through Rac1 signaling cascade. However, silencing WAVE2 with and without PlGF, did not have an additional effect on PAK1 activity levels (Fig. 4a–e, **p < 0.01), highlighting WAVE2 downstream activity to be independent of PAK1 in modulating actin nucleation.

**Fig. 5:**
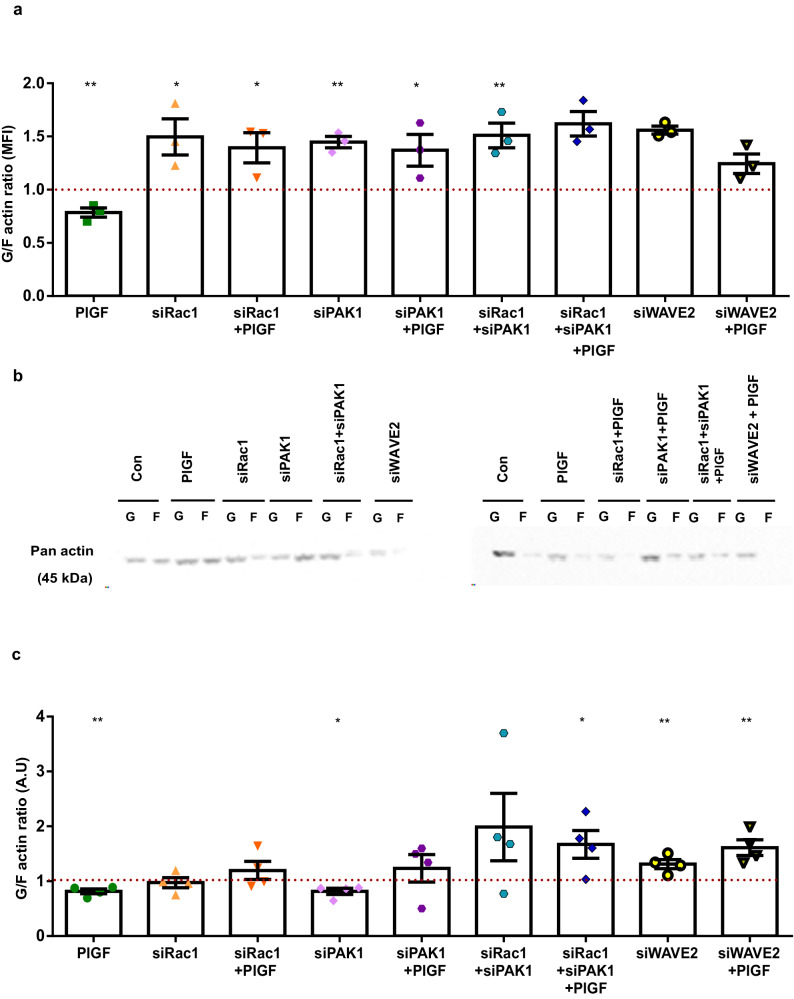
Effect of PlGF on endometrial actin dynamics under the influence of siRac1 siPAK1 and siWAVE2. **a** Arithmetic means ± SEM of G-actin over F-actin ratio in stromal cells after 6 days treatment with PlGF ± siRNAs transfections (*n* = 3, *p < 0.05, **p < 0.01). **b** Representative original western blot of soluble G-actin and filamentous F-actin in human endometrial stromal cells after 6 days of treatment with PlGF ± siRNAs transfections. **c** Arithmetic means ± SEM of G-actin over F-actin ratio in stromal cells after 6 days treatment with PlGF ± siRNAs transfections (*n* = 4, *p < 0.05 **p < 0.01). All the above data represented here is normalized to control cells. The control is represented as dotted lines in the graphs. The statistical significance was tested with an Unpaired t-test with Welch’s correction.

We next explored whether loss of Rac1/PAK1 or WAVE2 can affect G/F actin. Strikingly, silencing of Rac1/PAK1 or WAVE2 with and without PlGF was also followed by a significant increase of soluble G-actin over filamentous F-actin (flow cytometry; Fig. 5a, *p < 0.5, ***p < 0.001). This effect on reverting G and F actin ratio levels with siRNAs with or without PlGF was also evidenced with Western blot analysis measuring the pan actin levels of G and F actin protein lysates (Fig. 5b, c and Supplementary information, *p < 0.5, ***p < 0.001). Thus, the above-presented results confirm the inhibitory effect of siRac1, siPAK1, and siWAVE2. Further, it validates the PlGF-mediated activation of Rac1-PAK1 signaling axis and its downstream effect on endometrial actin dynamics.

### Pravastatin reverses PlGF-induced endometrial actin dynamics and improves BeWo cell invasion

**Fig. 6:**
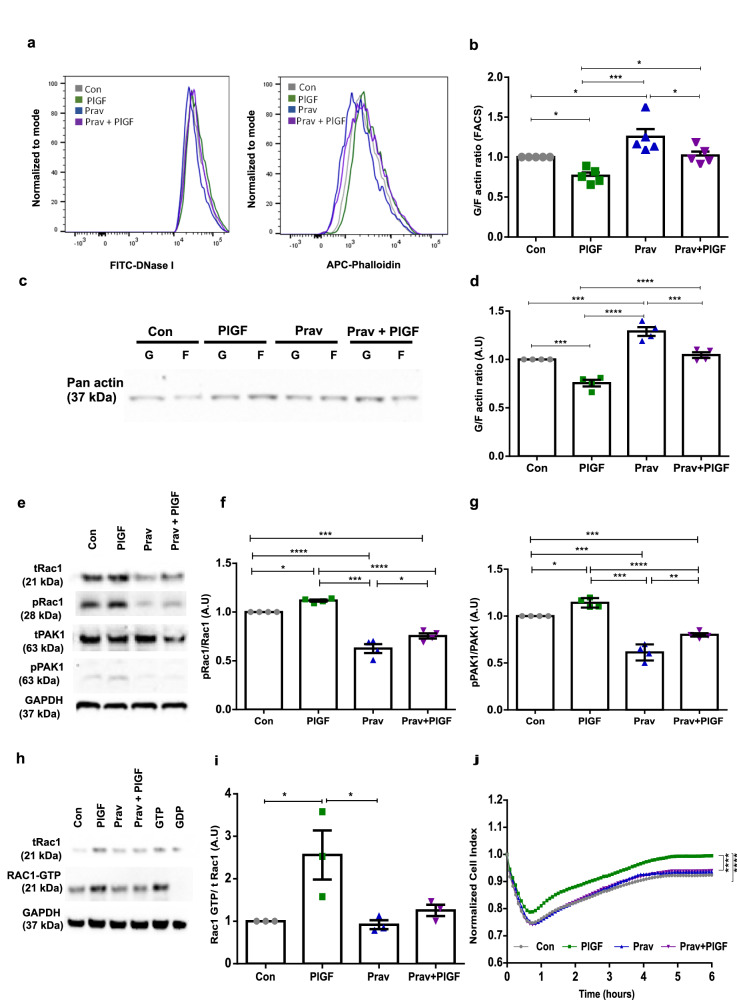
Pharmacological inhibition of PlGF-induced Rac1 activation with pravastatin. **a** Representative original histogram of DNaseI (G-actin; Left) and Phalloidin (F-actin; Right) binding in EnSCs after 6 days of treatment with PlGF ± pravastatin (10 mM, 24 h). **b** Arithmetic mean ± SEM of G-actin over F-actin ratio in EnSCs after 6 days treatment with PlGF ± pravastatin (24 h) (*n* = 5, *p < 0.05, ***p < 0.001). **c** Representative original western blot of soluble G-actin over filamentous F-actin in human EnSCs after 6 days treatment with PlGF ± pravastatin (24 h). **d** Arithmetic mean ± SEM of G-actin and F-actin ratio in stromal cells after 6 days treatment with PlGF ± pravastatin (24 h) (*n* = 4, ***p < 0.001, ****p < 0.0001). **e** Original western blots of total and phosphorylated levels of Rac1 and PAK1 targets with GAPDH used as loading control. **f** Arithmetic mean ± SEM of protein ratio of phosphorylated Rac1/ total Rac1 in total lysate of endometrial stromal cells treated with PlGF± pravastatin (24 h) (*n* = 5, *p < 0.05, ***p < 0.001, ****p < 0.0001). **g** Arithmetic mean ± SEM of *p*rotein ratio of phosphorylated PAK1/total PAK1 protein levels in total lysate of endometrial stromal cells treated with PlGF± pravastatin (24 h) (*n* = 5, *p < 0.05, **p < 0.01, ***p < 0.001, ****p < 0.0001). **h** Original western blots of total Rac1, Rac1 GTP, and GAPDH from Rac1 pull-down assay. **i** Arithmetic mean ± SEM of protein levels of activated form of Rac1 as Rac1GTP normalised to total Rac1in total lysate of endometrial stromal cells treated with PlGF± pravastatin (24 h) (*n* = 3, *p < 0.05). All the above data represented here is normalized to control cells. The statistical significance was tested with an Unpaired *t*-test with Welch’s correction. **j** Normalized cell index values of EnSCs monolayer to BeWo addition time point, cell index refers to the resistance in EnSCs layer post BeWo invasion for 6 h. EnSCs were under 6 days of treatment with PlGF ± pravastatin (24 h) (*n* = 3, ****p < 0.0001). The statistical significance for EIS analysis was tested at 5 h when the impedance was almost reaching a threshold, with an Unpaired *t*-test with Welch’s correction.

We next examined if PlGF-induced Rac1 activation and altered actin dynamics in EnSCs could be pharmacologically reversed. Therefore, we assessed the activity of pravastatin drug on Rac1 mechanism with and without PlGF treatment. We show pravastatin reverts back to the effect of PlGF on actin levels by increasing G and F actin ratio levels, as evidenced by both flow cytometry and Western blotting respectively. Taken together these results confirm that PlGF-treated EnSCs can be recused after a 24 h treatment with pravastatin, with levels almost reaching control (untreated) levels (Fig. 6a–g and Supplementary information), *p < 0.5, **p < 0.01, ***p < 0.001, ****p < 0.0001). Similarly, we also show an inhibitory effect of pravastatin on PlGF-induced Rac1-GTP (Fig. 6h–I and Supplementary information) levels.

Tight junctions, which connect adjacent cells, control the barrier of a tissue layer. PE is characterized by poor EVT invasion through the stroma to the spiral arteries^30^. We hypothesised that PlGF can impede normal invasion of BeWo through EnSCs. To test this, we used electrical impedance spectroscopy (EIS), which can measure (electrical) resistance which reflects the tightness of the junctions. To this end, EnSCs monolayers were formed in the presence or absence of PlGF for 6 days with or without 24 h pravastatin (10 µM) treatment. Following this BeWo cells were added and the invasion was monitored with EIS.

We show that the resistance of PlGF-treated stromal monolayer was higher compared to the control pointing to a reduced BeWo invasion (Fig. 6j, ***p < 0.0001). Strikingly, the reduced BeWo invasion in PlGF-treated EnSCs was reversed by pravastatin treatment (Fig. 6j, ****p < 0.0001). Collectively, these results indicate a clear beneficial effect of pravastatin in inhibiting PlGF-mediated Rac1 activation and normalising actin levels with improved BeWo cell invasion through the endometrial cells.

### Pravastatin reverts the PlGF-modified cell and ECM proteome in endometrial stromal cells

**Fig. 7:**
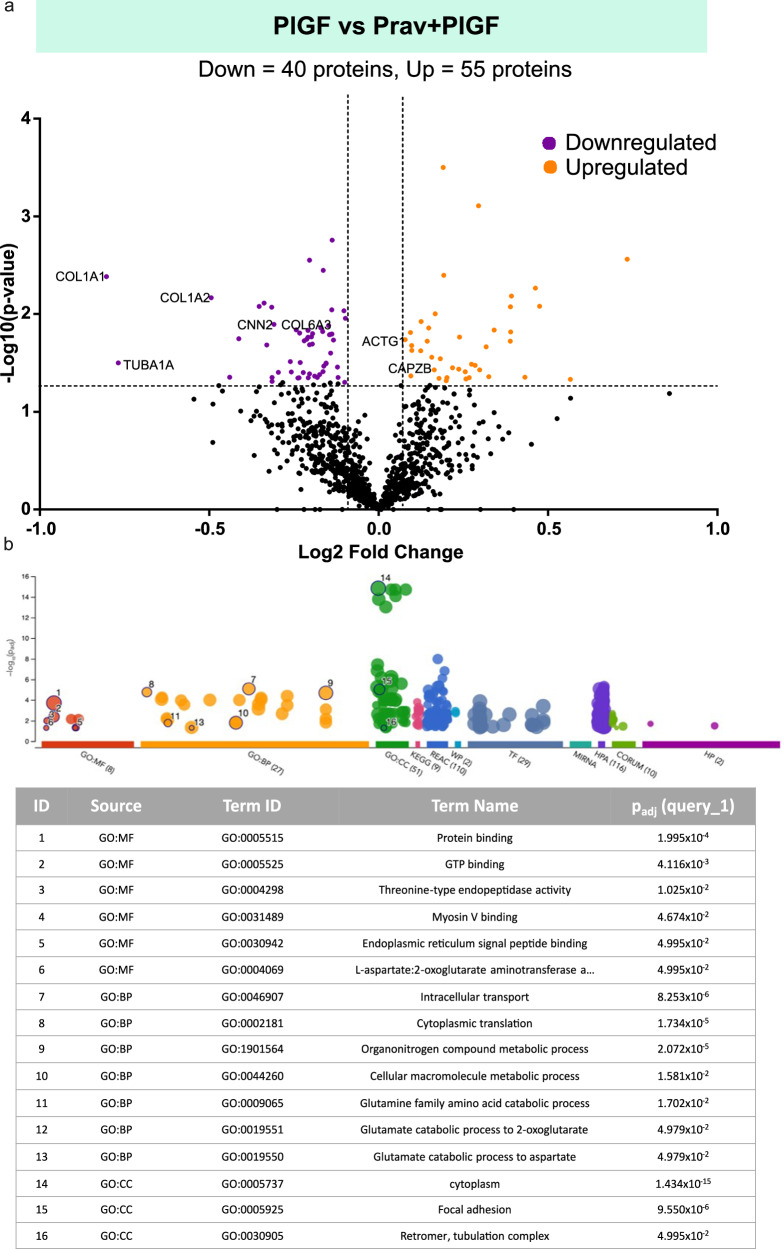
Mass spectroscopy analysis for the effect of pravastatin on PlGF-induced protein machinery. **a** Volcano plot showing upregulated (orange) and downregulated (violet) proteins in PlGF + pravastatin (n = 3) treated EnSCs compared to PlGF treatment (*n* = 3). Each point represents one protein; black points are the rest of the proteins obtained in the global proteomic analysis. The significance threshold range is 0.05 and the fold change threshold is -1 and +1. **b** Gene Ontology analysis of the protein signature associated with PlGF + pravastatin treatment in EnSCs shows enriched biological processes.

To investigate the comprehensive effect of PlGF and drug pravastatin in EnSCs, we utilized a quantitative proteomic approach. Proteomic analysis was performed by comparing protein expression patterns in PlGF (*n* = 3) and PlGF + pravastatin (*n* = 3) treated EnSCs. This comparison revealed a total of 95 dysregulated proteins. Differentially regulated proteins were shown in volcano plots as seen in Fig. 7a. A total of 55 upregulated (orange) and 40 (violet) downregulated differentially regulated proteins were identified as being associated with pravastatin treatment in PlGF-treated EnSCs. The heatmap describes the differentially regulated proteins expressed in different treatment groups (Con/PlGF/Prav/PlGF+Prav) in EnSCs following global proteomic analysis (Fig S11). Some of the upregulated proteins were involved in actin cytoskeleton regulation and stabilization such as ACTG1 and CAPZB. Some of the downregulated proteins included actin cytoskeleton and extracellular matrix remodelling proteins such as CNN2, COL1A1, COL1A2, COL6A, and TUBA1A. Gene Ontology analysis of the protein signature associated with pravastatin treatment in PlGF-treated EnSCs identified pathways associated with GTP binding, intracellular transport and cytoplasmic translation (Fig. 7b). Thus, the proteomic analysis pointed to a reversal of actin regulation, extracellular matrix remodelling mechanics and cell-stiffness with the absence of GTPases pathway activity with pravastatin treatment on PlGF treated EnSCs.

## Discussion

PE is a disorder that affects 3–5% of pregnant women and is a leading cause of maternal and neonatal mortality and morbidity^33,34^. The principal features include a new onset of hypertension and proteinuria. Currently, the only definitive cure is delivery.

The pathogenesis of PE is multifactorial and is incompletely understood but appears to be a consequence of maternal uteroplacental malperfusion and abnormal adaptive placentation^35,36^. Recent studies suggest that PE might in fact be a disorder of the decidua prior to pregnancy^3,8^. These authors demonstrated in vitro that EnSCs isolated from patients with a history of PE failed to decidualize and that a decidual molecular defect is an important contributor in PE, suggesting a pivotal role of the maternal decidua in the development of PE pathogenesis. However, this study did not provide any molecular mechanism causing defective decidua.

The well-established marker for PE diagnosis in asymptomatic pregnant women is the use of the biomarker sFLT1/PlGF. The sFLT1/PlGF ratio shows a sensitivity of 66.2% and a specificity of 83.1%^18,37^. However, its use is effective only 4 weeks before PE symptoms manifest. Therefore, during early pregnancy, there is an absence of screening methods (both highly sensitive and specific) to diagnose PE earlier and therefore avoid mortality and morbidity. Indirect evidence points to a pathological role for PlGF in pregnancy^38–40^, but the role of endogenous PlGF on non-endothelial cells prior to pregnancy, specifically in modulating the decidua remains unknown.

To identify the involvement of altered cellular mechanics in the decidua in PE progression, we performed manual mining of bulk RNA-seq from endometrial biopsies (*GSE172381*)^8^. 593 genes were identified to be differentially expressed in the decidua from patients with a history of PE compared to the women with healthy pregnancies. Remarkably, according to gene enrichment analysis, actin dynamics and cytoskeleton signalling pathways were highly dysregulated. Thus, the altered expression of extracellular matrix, cell motility, cell component organisation, and cytoskeletal components, prior to pregnancy could disturb the microenvironment at the maternal interface during early pregnancy and placentation contributing to the development of PE.

**Fig. 8:**
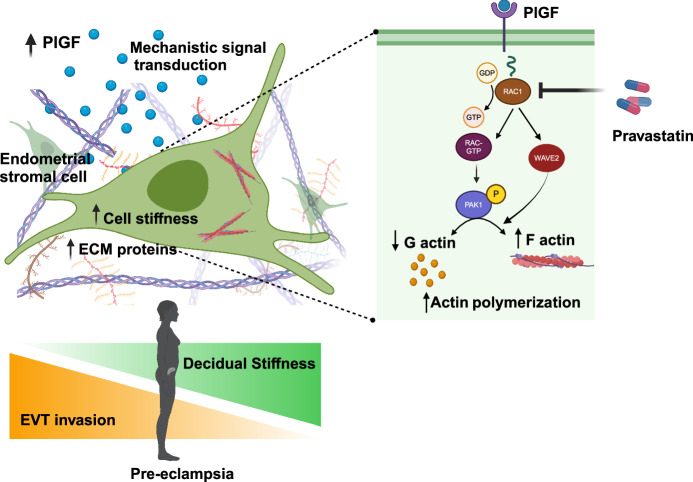
Graphical abstract describing the effect of pathological PlGF levels in altered endometrial cytoskeletal dynamics. Excessive endometrial PlGF promotes Rac1 activity by modulating Rac1-GDP conversion to Rac1-GTP in EnSCs. Rac1 activation upregulates its downstream targets PAK1 and WAVE2, leading to alterations in G and F-actin organisation and turn over with an effect on an increase in cell stiffness. High levels of PlGF also alter the cell’s microenvironment with an increase in ECM-associated proteins altering endometrial biomechanics at a global cellular level. Dysregulation of endometrial/decidual mechanobiology with an increase in cell and ECM stiffness will impede EVT invasion during early pregnancy, leading to shallow placentation as seen in pre-eclampsia. Excessive PlGF-induced cell stiffness dysregulation in the endometrium could be pharmacologically reversed with a potential therapeutic target, pravastatin. The figure was created using BioRender.

The main aim of this study has been to investigate the effect of PlGF on endometrial cellular mechanics. Our study revealed that PlGF affect cellular actin regulation and ECM machinery at a global level employing proteomics. Further, PlGF influences stromal cells showed a variety of protein cargo, including cell signalling molecules, factors that maintain cell polarity, intracellular transport mediators, and actin-modulating proteins such as Rho GTPases activating protein. Rac1, a member of the Rho family of small GTPases controls a wide array of cellular functions. In response to diverse signals, it converts from an inactive GDP-bound form to an active GTP-bound form. Rac1 signalling has been directly implicated in the regulation of cell motility, such as migration and invasion^41–43^, and is therefore an important regulator of actin cytoskeleton organization^44^. In our study, we show that following PlGF treatment there is a significant up-regulation of Rac1 activity and even accounts for the observed actin reorganization, followed by cell stiffening. Rac1 activation is further recognised to regulate actin polymerization interacting with the Arp2/3-mediated actin nucleation pathway through its target WAVE2 to improve actin nucleation. In our study however, we observed a PlGF-induced increase of Rac1 phosphorylation a regulator of both PAK1 and WAVE2, indicating activation of both of these signalling effectors in endometrial stromal cells. In line with this, we observed the reorganization of the actin filaments network towards the polymerization of F-actin filaments. Since modification of actin dynamics through polymerization has been reported to account for filaments of higher mechanical stability^45^, our observations on cell stiffening in response to PlGF treatment may directly be correlated to the observed PlGF-induced actin depolymerization. Moreover, siRNA targeting either Rac1, PAK1, and WAVE2 or in combination with PlGF did not increase these targets. This observation confirms that PlGF is a target of the Rac1/PAK1 signalling cascade (Fig. 8).

Cell biomechanics is relevant for all tissues and the stiffness of most human tissues diverges greatly from the brain (soft, ~0.2 kPa) to bone (rigid, ~10^6^ kPa)^46^. The decidua (biopsies) has a stiffness of around 1.2 kPa^47^. During early pregnancy, EVTs invade and congregate around the decidua and induce the remodeling of the spiral arteries. Modifications in stiffness within the endometrium could thereby impact EVT invasion, and the most notable change in stiffness that they will encounter is when only they approach the myometrium, the smooth muscular layer beneath the decidua. It is interesting to point out that the stiffness of smooth muscle tissue is ~5 kPa, five times that of decidua, which is similar to the stiffness observed post-treatment with PlGF in EnSCs (4.9 kPa). Once EVT cells arrive at the (inner third) myometrium, they halt their migration and differentiate into multinucleated giant cells. Taken together, we speculate that, prior to pregnancy, increased levels of PlGF in the endometrium could result in an under-invasion of EVT pathology in decidua due to the enhanced stiffness. Increased stiffness in the endometrium hence interferes with the decidualization function of the stromal cells and prevents or hampers EVT migration, therefore preventing effective remodelling of spiral arteries, resulting in PE pathogenesis later on in pregnancy.

Statins (HMG-CoA reductase inhibitors) are widely used and are effective in preventing cardiovascular mortality and morbidity^48^. In rodent models of PE, pravastatin administered in early pregnancy was able to restore angiogenic imbalance and re-establish normal endothelial function^49,50^. These preliminary animal studies provide a strong rationale for the use of pravastatin in the prevention of PE in humans. It is noteworthy to point out two recent clinical trials using pravastatin. In 2021, Döbert et al., performed a double-blind, placebo-controlled trial in 1120 women^51^. Women screened for term PE were given 20 mg daily dose of pravastatin for 35–37 weeks gestation until delivery. They found no difference between the pravastatin and placebo group. In, another study performed during the early phase of pregnancy (12–16 weeks) women were given 10 mg per day pravastatin until delivery^52^. Although the numbers were limited, this study showed a significant reduction in incidence and severity of PE when pravastatin was administered earlier in pregnancy during EVT invasion and thus placental formation. The use of statins in pregnancy is highly debated because of its possible teratogenicity^53^. However recent studies suggest that statin exposure during pregnancy is not associated with an increased risk of congenital anomaly in humans^53,54^. The above-mentioned retrospective clinical trials with use of pravastatin for PE prevention or treatment also showed no identifiable safety risks associated with pravastatin in their cohorts^51,52^. Whilst this clinical trial is very encouraging, further studies are warranted to support the necessity of statins for the treatment and prevention of PE pathogenesis and possible teratogenicity if prescribed in early pregnancy.

Rac1 is post-translationally modified by an isoprenyl lipid group, specifically geranylgeranyl diphosphate (GGPP), which is thought to regulate its subcellular localization^55,56^. Statins are known to inhibit mevalonate synthesis upstream of both cholesterol and GGPP synthesis, and thus, statins can inhibit the isoprenylation of cytoskeletal regulator Rac1^57^. Studies also support a potential link between statins and actin modulation^58–61^. Statins are reported to affect the rearrangement of F-actin with a regulatory effect on cofilin, an actin-binding protein involved in the control of cell shape and motility^60^. Similarly, pravastatin and simvastatin are known to downregulate pY14-caveolin, which indicates a loss of actin interactions^58^. Recently, Sarkar et al. reported that lovastatin induces significant polymerization of the actin cytoskeleton in regulating the dynamics of the serotonin1A receptor^59^. Thus, playing a major role in the modulation of cognitive and behavioural functions^59^. In our study, we report an inhibitory effect of pravastatin on Rac1 activation, reverting actin polymerization to normal levels and improving trophoblast cell invasion through EnSCs monolayer. Additionally, our proteomic analysis confirms this inhibitory action of pravastatin on PlGF-treated EnSCs, with downregulating a few actin-regulating targets, ECM modulating proteins, and reversing GTPases activity. Thereby, we postulate that pravastatin could be a potential pharmacological candidate to reverse altered Rac1 signaling and further could potentially reduce decidual cell stiffness, allowing proper EVT invasion into the maternal microenvironment, thus preventing shallow placentation which is the main driver of PE.

However, given the non-cholesterol pleiotropic effects of statins such as reduction of inflammatory mediators, inhibiting the T-helper cell immune responses or nitric oxide synthases^62,63^, which in turn may impact on decidualisation and pregnancy itself, well-planned research is required to validate its use in humans. Despite that, these studies demonstrate the equipoise necessary to approve a trial of prophylactic pravastatin either prior to pregnancy and/or in early pregnancy to prevent PE.

In conclusion, this study highlights an underlying transcriptomic defect of cytoskeletal-actin dynamics (using publicly available data sets) that may explain the maternal role of shallow or impaired placentation seen in PE. The present study further describes a previously uncharacterised function of PlGF in upregulating the expression and activity of the small G-protein Rac1 and of the kinase PAK1 with subsequent actin polymerization as well as the increase of cell stiffness. Our work is an important step towards the development of novel approaches that facilitate an early evaluation of the risk for PE and to treat this detrimental disease. Thus, this unique role of PlGF may provide avenues to pursue new therapeutic agents or non-invasive strategies capable of curtailing pregnancy disorders such as PE.

## Methods

### Cell lines

Human EnSCs (#T0533, Applied Biological Materials Inc)^23,64,65^ were cultured at 37 °C in a humidified 5% CO_2_ atmosphere in DMEM/F-12 medium (#11039-021, Invitrogen) containing 10% (v/v) dextran-coated charcoal striped (#C6241, Sigma) fetal bovine serum (#10270-106, Invitrogen). Human BeWo cells (#86082803, Sigma)^66,67^, a human trophoblast cell line, were cultured in DMEM/F-12 medium (#11039-021, Invitrogen) containing 10% (v/v) fetal bovine serum (#10270-106, Invitrogen).

### Cell culture treatment and transfection

EnSCs were subjected to treatment with PlGF (#P1588, Sigma) at a concentration of 20 ng/ml for 6 days^68^. For decidualization, EnSCs were treated with 0.5 μM 8-Bromoadenosine-3’,5’-cyclic monophosphate sodium salt (cAMP) (#1140, Tocris) and 1 μM Medroxyprogesterone 17-acetate (MPA) (#M6013, Sigma) for 6 days^69^. The cell culture medium was replaced every 48 h with fresh treatment media. The experimental groups are indicated as control (untreated EnSCs), PlGF, cAMP+MPA, and cAMP+MPA+ PlGF.

Where indicated, EnSCs were treated with pravastatin (Pravastatin sodium salt hydrate, #P4498, Sigma) with or without PlGF treatment at 10 µM for 24 h^70^. The experimental groups are classified as control (untreated EnSCs), PlGF, pravastatin (Prav), and pravastatin + PlGF (Prav + PlGF). Epidermal Growth factor (EGF) was used as a positive control for Rac1 activation. (#324831, Sigma) at a concentration of 100 ng/ml for 24 h^71^.

For gene silencing, EnSCs were treated with siRNAs^72^, such as siRac1 (50 nM, #L-003560-00-0010, Dharmacon), siPAK1 (20 µM, #L-003521-00-0010, Dharmacon), and siWAVE2 (5 nM, #s55787, ThermoFisher Scientific). The siRNAs were transfected with Lipofectamine RNAiMAX (#13778075, ThermoFisher Scientific) for 48 h with and without combination with PlGF treatment.

### Preparation of cell lysate for proteomic analysis

For proteome analyses, cell suspensions were prepared from EnSCs treated with and without PlGF(20 ng/ml for 6 days). Cells were lysed by adding lysis buffer [5% 1 M Tris/HCl pH 7,4, 2% 5 M NaCl, 1% Triton X 100, 1% PMSF, 4% protease inhibitor cocktail (Sigma-Aldrich, St. Louis, USA)] on ice. Ten micrograms of each sample were digested in solution with trypsin. After desalting using C18 stage tips, extracted peptides were separated on an Easy-nLC 1200 system coupled to a Q Exactive HFX mass spectrometer (Thermo Fisher Scientific). The peptide mixtures were separated using a 90 minutes segmented gradient from 10-33-50-90% of HPLC solvent B (80% acetonitrile in 0.1% formic acid) in HPLC solvent A (0.1% formic acid) at a flow rate of 200 nl/min. The 12 most intense precursor ions were sequentially fragmented in each scan cycle using higher energy collisional dissociation (HCD) fragmentation. Acquired MS spectra were processed with MaxQuant software package version 1.6.7.0 with integrated Andromeda search engine^73^ Database search was performed against a target-decoy Homo sapiens database obtained from Uniprot, containing 103.859 protein entries and 286 commonly observed contaminants. Peptide, protein, and modification site identifications were reported at a false discovery rate (FDR) of 0.01, estimated by the target/decoy approach. The LFQ (Label-Free Quantification) algorithm was enabled, as well as match between runs and LFQ protein intensities was used for relative protein quantification. Data analysis was performed using Perseus^74^ and STRING v11 database.

### Quantitative Real-time PCR (qRT-PCR)

Total RNA was extracted from EnSCs cultures using Trizol (#15596026, Invitrogen) based on a phenol-chloroform extraction approach. Equal amounts of total RNA (1 μg) were reverse transcribed by using the Maxima h Minus method (#M1681, ThermoFisher Scientific), and the resulting cDNA was used as a template in qRT-PCR analysis. The gene-specific primer pairs were designed using the Primerblast (NCBI) software and purchased from Sigma. Detection of gene expression was performed using the PowerUp SYBR Green Master Mix (#A25742, ThermoFisher Scientific), and quantitative RT-PCR was performed on a QuantStudio 3 Real-Time PCR system (#A28567, ThermoFisher Scientific). L19 was used to normalize variances in input cDNA. The expression levels of the samples are given as arbitrary units defined by the ^ΔΔ^Ct method. All measurements were performed in triplicate. Melting curve analysis confirmed the amplification specificity of the genes. Sequences of human primers used for qRT-PCR are available on request.

### Western Blotting

Whole cell protein lysates were extracted from EnSCs using hot 1X Laemmli buffer (final concentration) with a cell scraper post treatment^23^. Protein lysates were then loaded onto 4-12% Bis tris mini protein gels (#NW04120BOX, ThermoFisher Scientific) using the XCell SureLock® Mini-Cell apparatus (Invitrogen) followed by electrophoresis. The protein from the gel was then transferred onto a nitrocellulose membrane (#GE10600003, Amersham Biosciences) using a semi-dry transfer approach. After incubation with 5% non-fat milk in TBS-T (10 mM Tris, pH 8.0, 150 mM NaCl, 0.5% Tween 20) for 60 min, the membrane was washed twice with TBS-T and incubated with primary antibodies against Rac1 (1:1000, #2465 s, Cell Signaling Technology), phospho Rac1 (1:500, #2461 S, Cell Signaling Technology), PAK1 (1:500, 51137-1-AP, Protein Tech), phospho PAK1 (1:1000, #2601, Cell Signaling Technology), WAVE2 (1:1000,#3659 s, Cell Signaling Technology), ARP2 (1:1000, #3128 s, Cell Signaling Technology), ARP3 (1:1000,#4738 s, Cell Signaling Technology) or GAPDH (1:1000, #5174, Cell Signaling Technology) at 4 °C for overnight. The concentrations of the antibodies employed in the study are as per the manufacturer’s instructions.

Post overnight incubation with primary antibodies, membranes were washed with TBS-T and incubated with HRP-conjugated anti-rabbit secondary (1:2000, #7074 s, Cell Signaling Technology) antibody for 1 h in room temperature. Post-secondary antibody incubation, blots were washed with TBS-T and developed with the ECL system (#R-03031-D25, Advansta) according to the manufacturer’s protocols. The fluorescence signals were obtained with an iBright CL1000 (ThermoFisher Scientific), and the intensities were assessed by a densitometry analysis to measure the relative expression of the target proteins using GAPDH as a control by ImageJ software.

### Active Rac1 pull-down assay

Rac1 pull down assays were performed using a Rac1 Activity Assay kit (#16118, ThermoFisher Scientific) to monitor Rac1 small GTPase activation as per manufacturer instruction.

### Measurement of the G/F actin ratio by Triton X-100 fractionation

To quantify the effect of PlGF on actin polymerization in EnSCs, cells were incubated with actin extraction buffer and processed for G and F-actin protein lysates^22^. Bradford assay was performed to obtain protein concentration and equal volumes of each fraction were boiled with 1X Lamelli Buffer at 95 °C for 15 min. Proteins were separated on 10% SDS-polyacrylamide gels and transferred to PVDF membranes (#GE10600029, Amersham Biosciences). Non-specific binding sites were blocked by 1 h incubation at room temperature with 5% non-fat dry milk in TBS-T. The membranes were incubated overnight at 4 °C with primary antibodies against Pan-actin rabbit monoclonal antibodies (1:1000, #D18C11, Cell Signaling Technology). Post incubation with primary antibody, membranes were washed with TBS-T and incubated with HRP-conjugated anti-rabbit secondary (1:2000, #7074 s, Cell Signaling Technology) antibodies for 1 hour in room temperature. Antibody binding was detected post-developing with the ECL system (#R-03031-D25, Advansta) according to the manufacturer’s protocols. The fluorescence signals were obtained with an iBright CL1000 (ThermoFisher Scientific), and the intensities were assessed by a densitometry analysis to measure the relative expression of the pan- actin by ImageJ software.

### G/F actin ratio by Flow cytometry

To study the influence of PlGF treatment on G/F action ratio with flow cytometry, post treatment with PlGF (20 ng/ml for 6 days), EnSCs (≈1.0 × 10^5^ cells) were first fixed with 4% paraformaldehyde (PFA) and then permeabilised with 1x Permeabilization buffer (#00-8333-56, eBioscience) and subsequently stained with 10 µl of fluorescent DNAse1-Alexaflour-488 (#D12371, Thermofisher Scientific) (5 mg/ml) for detection of G-actin and 1 µl fluorescent Phalloidin-eFluor® 660 (1000x) (1:1000, #50655905, Thermofisher Scientific) for detection of F-actin. The quantitative measure of the respective fluorescent labels was measured using green (FL-1) and red channel (FL-4) on a BD LSRFortessa™ Cell Analyzer (BD Biosciences) and the analysis was performed using Flowjo software (Flowjo LLC, USA). The ratio of G/F is calculated from the geometric mean values.

### Immunofluorescence

For immunolabelling of cells, EnSCs (5000 cells) were seeded on glass chamber slides (#94.6170.402, Sarstedt) and cultured in 10% DCC FBS containing DMEM medium. Post treatment with PlGF (20 ng/ml for 6 days) in 2% DCC FBS medium, the cells were fixed with 4% paraformaldehyde for 15 min, washed with PBS, and permeabilized for 15 min in 0.1% Triton X-100/PBS. The samples were then blocked with 5% BSA in 0.1% TritonX-100/PBS for 1 h at RT and washed with PBS. The cells were then stained for F-actin with eflour 660-phalloidin (1:1000, #50655905, ThermoFisher Scientific) for 30 min at RT. Post incubation, slides were washed again with PBS, dehydrated, air-dried, and mounted using ProLong Gold antifade reagent containing DAPI (#P36931, Invitrogen). Fluorescence was detected with LSM 800 confocal laser scanning microscope (Zeiss). The images were captured using oil immersion, 40x objective lens. Scale bar - 20 µm. Mean fluorescence intensities were calculated using ImageJ software.

### Atomic force microscopy (AFM)

AFM experiments on live EnSCs were carried out with a commercial AFM setup (MFP3D, Asylum Research, Santa Barbara, USA) with cells seeded on culture dishes at a density of 5×10^4^ cells/cm². EnSCs were treated with either 20 ng/ml PlGF, decidualization treatment (cAMP+MPA), or 100 ng/ml EGF as a positive control for 6 days or 24 h, respectively, in a 2% DCC FBS treatment medium.

Additionally, AFM measurements were performed on cells seeded onto a polydimethylsiloxane (PDMS) substrate as described in Kenry et al.,^75^(CY52-276, Dow Corning, Toray, Japan). The two components, A and B, were mixed for 15 min at a weight ratio of 6:5. The mixture was then degassed in a vacuum chamber, poured into Petri dishes, and cured in an oven at 80 °C for 12 h, to yield soft substrates with a bulk elastic modulus of 1–3 kPa^75^. EnSCs were seeded on these soft substrates at a density of 2×10^4^ cells/cm^2^ and were subjected to PlGF or/and decidualization treatment as described above.

Single EnSCs were imaged with AFM in the force mapping mode. To study cell stiffness, maps of 10×10 force indentation curves were recorded on a 5 × 5 μm² scan area on the central region of cells using a pyramidal tip (Bio-MLCT, cantilever C, spring constant 0.01 N/m, side angle 35°, Bruker AXS S.A.S., Champs-sur-Marne, France). The maximum deflection was set to 100 nm, resulting in an indentation depth of typically 400–600 nm, which is much smaller than the cell height, thereby an influence of the underlying stiff substrate on the measured Young’s modulus was avoided^76^. Additionally, to display the whole cell body, we performed maps of 30 × 30 or 50 × 50 force indentation curves on an 80 × 80 µm^2^ or 90×90 µm^2^ scan area. Force-indentation curves were fitted with the pyramidal Sneddon model^77^ to calculate the Young´s modulus, E. Data were analysed with the data analysis package Igor Pro (Wavemetrics, Lake Oswego, OR, USA). The mean value of all Young´s modulus values on a cell was considered representative of that cell.

### Electrical Impedance spectroscopy (EIS)

The invasion of BeWo through the stromal monolayer was studied with EIS measurements. The E-16 plates were coated with 0.1% gelatin and let to incubate for an hour at 37 °C. Next, 100 µl of 2% DCC FBS DMEM was added to E16 plate for background measurement. The plate was mounted onto the xCELLigence RTCA (Agilent, Germany) for background reading. Later, EnSCs were trypsinized and 3000 cells per well were added to the E16 plates and kept for incubation at 37 °C for cells to equilibrate and adhere. Post 24 h, the cells were treated with respective treatment conditions (Con/PlGF/Prav/Prav+PlGF) as mentioned above for 6 days. Ten hours prior treatment endpoint, E16 plates containing cells were clamped again onto the xCELLigence RTCA and placed in the incubator at 37 °C. Live cell impedance was monitored every 15 min for a total period of 10 h to ensure stromal cell monolayer formation.

Post treatment time point, the cell index measurement was paused and 2500 BeWo cells per 100 µl of 10% FBS DMEM were added. EIS cell index measurement was continued to monitor to the BeWo invasion through EnSCs monolayer. Live cell impedance was monitored every 5 min for a total period of 6 hours. Data is represented as normalized cell index relative to the time point BeWo cells were added to the EnSCs monolayer. Data acquisition and data analysis was performed using RTCA Software Pro (Agilent, Germany).

### Wound healing scratch assay

EnSCs were seeded in a six-well plate at a concentration of 200 × 10^3^ cells per well. After reaching 100% confluency, the wells were scratched in the centre vertically with a 200 μL tip to create a wound area. Cells were treated with either PlGF (20 ng/ml) for 6 days or/and thymidine (2 mm; #T1895, Sigma) for 42 h or remained untreated. Pictures were then taken immediately at 0 h using the EVOS M7000 cell imaging system (ThermoFisher Scientific) at an objective 4X. The cells were then allowed to incubate at 37 °C in a humidified 5% CO_2_ atmosphere with the treatment medium for the next 24 h. Post incubation, cell migration was monitored by taking pictures at 24 h. Assessment of percentage of wound healing was quantified using the ImageJ software and presented as fold change.

### BrdU cell proliferation assay

The effect of PlGF on EnSCs proliferation was measured using BrdU cell proliferation assay (#QIA58, Sigma). Briefly after the treatment of EnSCs with PlGF (20 ng/ml) for 6 days or/and thymidine (2 mm; #T1895, Sigma) for 42 h, cells were immunolabelled for BrdU and incubated for an additional 24 h. Incorporated BrdU was detected by the BrdU Cell Proliferation Assay as instructed in the manufacture protocol.

### In silico data analysis

In silico analysis was performed on open-access gene-expression data. To study the kinetics of PlGF expression levels across the menstrual cycle, we obtained analysis of the normal endometrium at distinct phases of the menstrual cycle (*GDS2052*). To investigate PlGF and PAK1 mRNA levels in term preeclampsia, we obtained PAK1 gene activity data in human decidua of preeclamptic women and healthy pregnant women (*GDS2548*). Lastly, to understand the maternal influence on PE pathogenesis, transcriptomic profiling of decidua from previous healthy and preeclamptic pregnancies were studied (*GSE172381*).

### Statistics and Reproducibility

Data are provided as means ± SEM, and *n* represents the number of independent experiments. Data provided here were tested for significance using student’s unpaired two-tailed t-test with Welch’s correction approach and nonparametric Mann-Whitney U test methods. PlGF treated samples were normalized to the control. Results with *p* < 0.05 were considered statistically significant. Figures and statistical analysis were made in Graphpad Prism (USA) or with R.

### Reporting summary

Further information on research design is available in the Nature Portfolio Reporting Summary linked to this article.

### Supplementary information

Supplementary Information (new)

Description of Additional Supplementary Files

Supplementary Data

Reporting Summary

## Data Availability

Proteomics data have been submitted to the PRoteomics IDEntification database (PRIDE) with accession number PXD050237. The source data behind the graphs in the figures can be found in Supplementary data 2, the uncropped, original Western blots are shown in Supplementary Fig. S12.
