# Supplementary material for: Excessive endometrial PlGF- Rac1 signalling underlies endometrial cell stiffness linked to pre-eclampsia
Source: Commun Biol. 2024 May 4;7:530. doi: 10.1038/s42003-024-06220-7 (PMC11069541; doi:10.1038/s42003-024-06220-7)

Excessive endometrial PlGF- Rac1 signalling underlies endometrial cell stiffness linked to pre-eclampsia.

Supplementary Figures

Fig S1

a

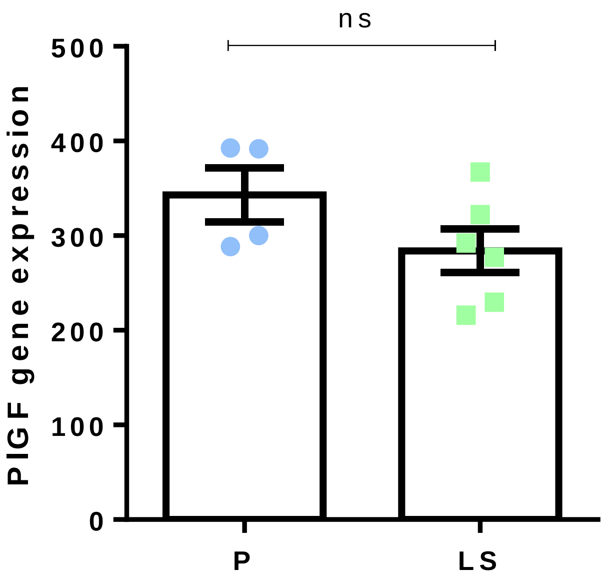

b

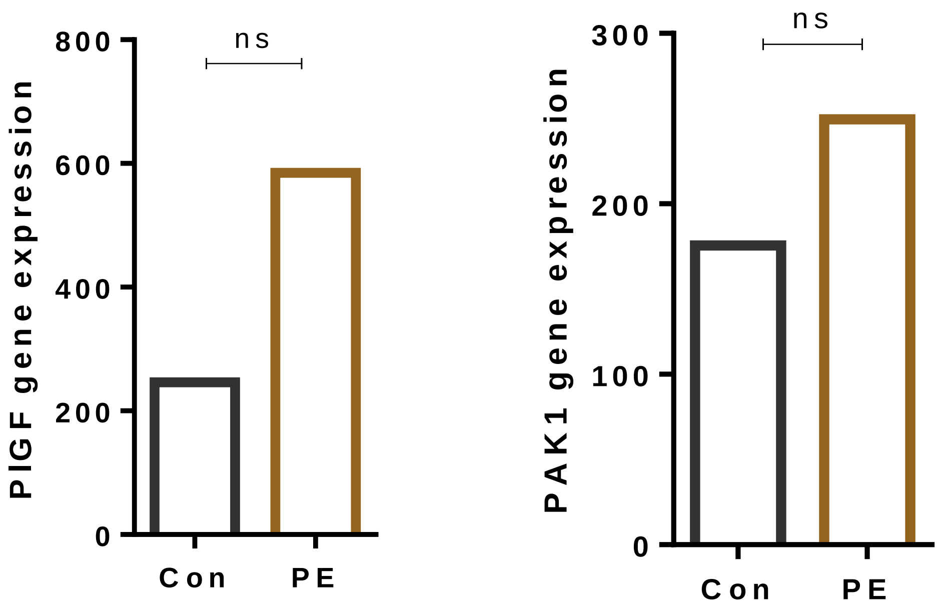

FigS1: a. Expression kinetics of PlGF across the menstrual cycle in the proliferative (P) phase (n=4) and late secretory (LS) phase (n=6) (*GDS2052*). Statistical significance was tested with unpaired t test and found to be non-significant. b. PlGF and PAK1 transcripts in term decidua's of preeclamptic women (n=10) compared to the decidua from healthy (n=10) pregnant women (*GEO2548*). The GEO dataset for the control and diseased samples were retrieved using the GEO2R query bioconductor package. Gene expression value was represented as average value pooled from all 10 samples respectively. Statistical significance was tested with unpaired t test. The gene expressions between control and PE are not statistically significant but show both upregulated PlGF and PAK1 gene expression pattern in PE decidua's compared to healthy pregnant tissue samples.

Fig S1

c

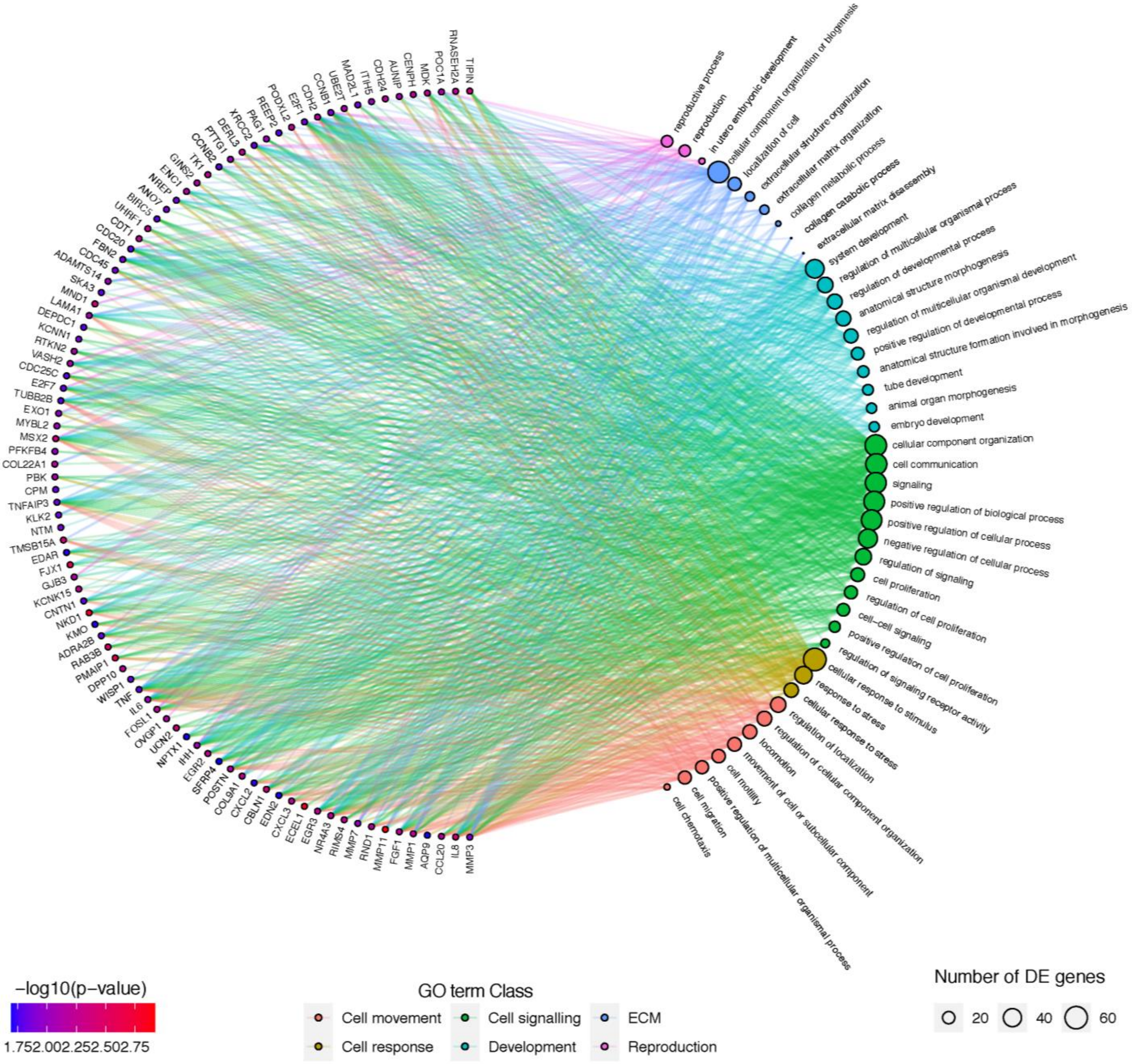

FigS1c. Gene ontology analysis from RNA sequencing array (RNA-seq, *GSE172381*) from endometrial biopsies obtained from women who had severe PE in a previous pregnancy (n = 24).

Fig S1

d

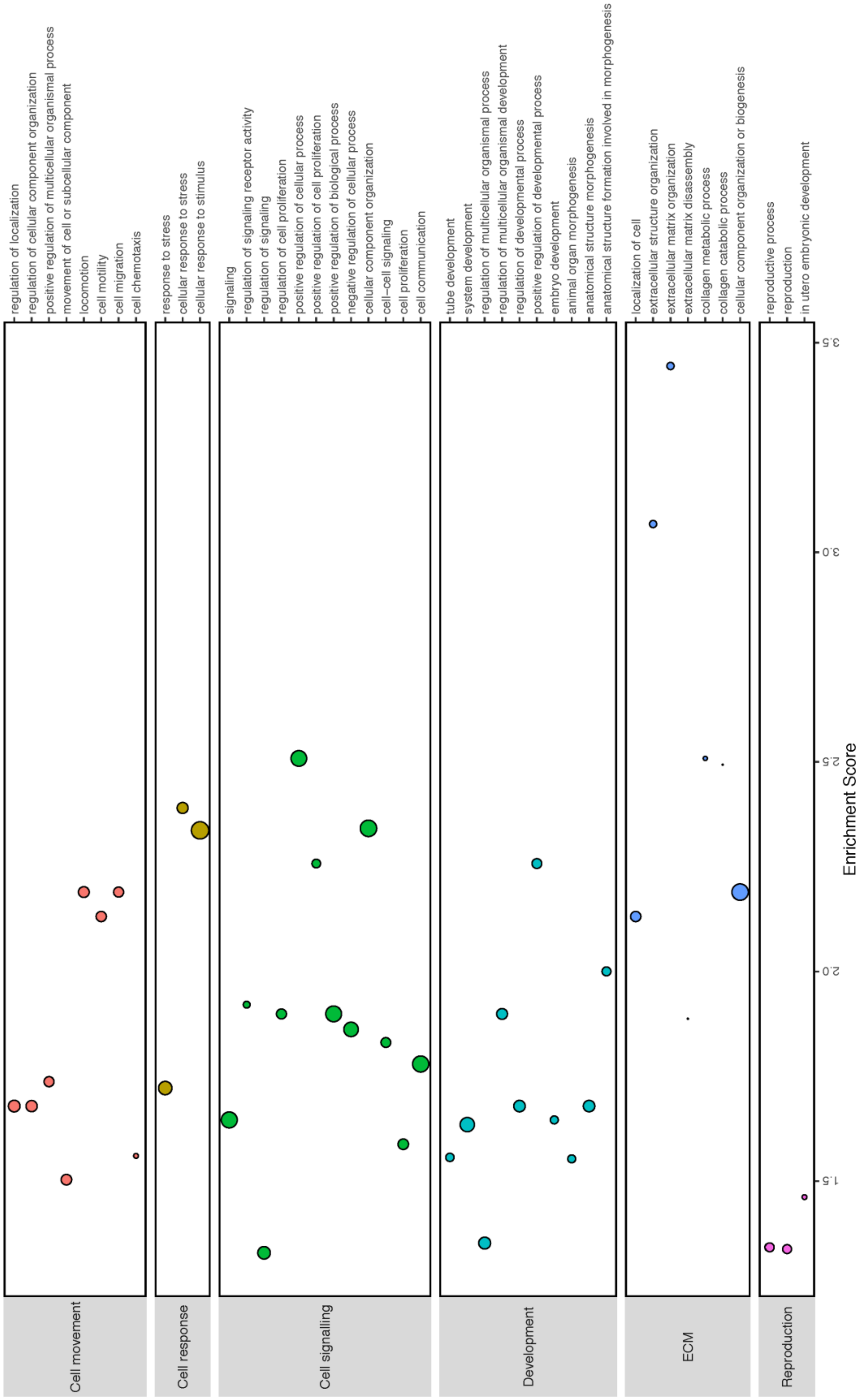

FigS1d: Enriched biological process from GO analysis from RNA sequencing array (RNA-seq, *GSE172381*) from endometrial biopsies obtained from women who had severe PE in a previous pregnancy (n = 24).

Fig S2

a

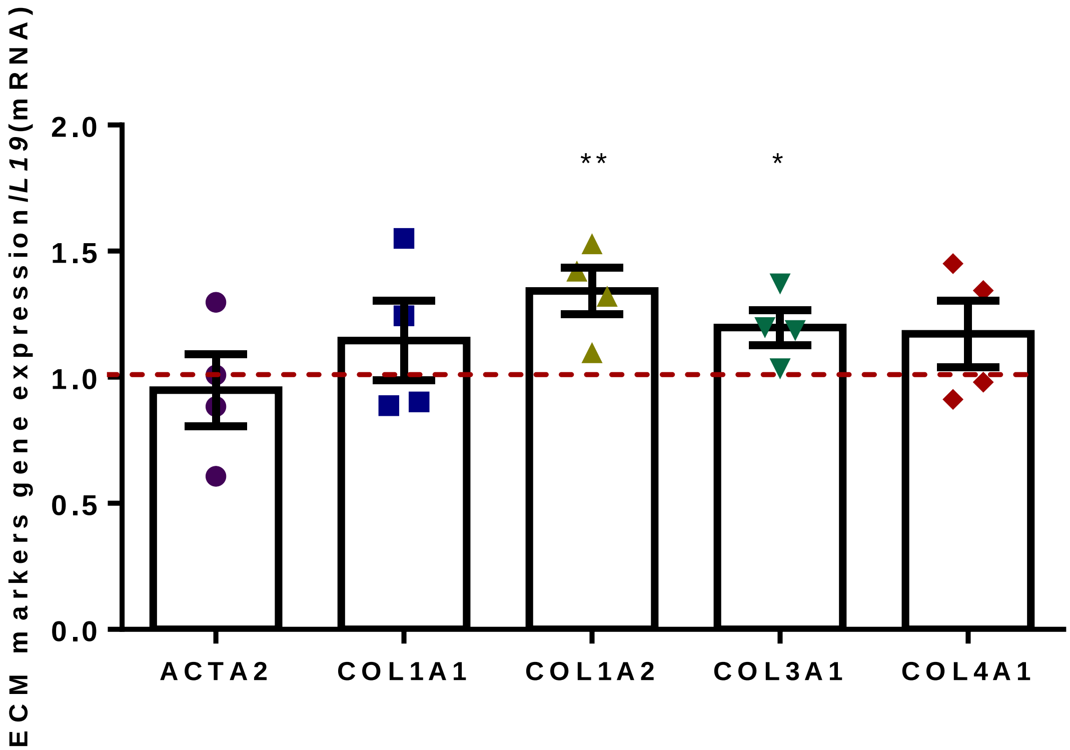

b

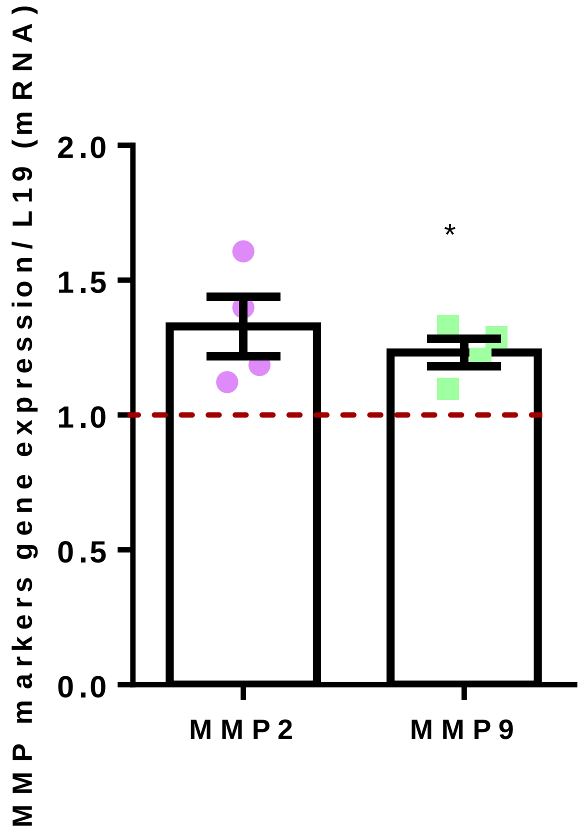

Fid S2: a) Arithmetic mean ± SEM of ECM markers transcript level in EnSCs on treatment with PlGF (n=4, *, p<0.05, **, p< 0.01). (b) Arithmetic mean ± SEM of MMP markers gene expression levels in EnSCs on treatment with PlGF (n=4, *, p<0.05). All the above data represented here is normalized to control cells. An unpaired t test with Welch's correction was used to test for statistical significance.

Fig S3

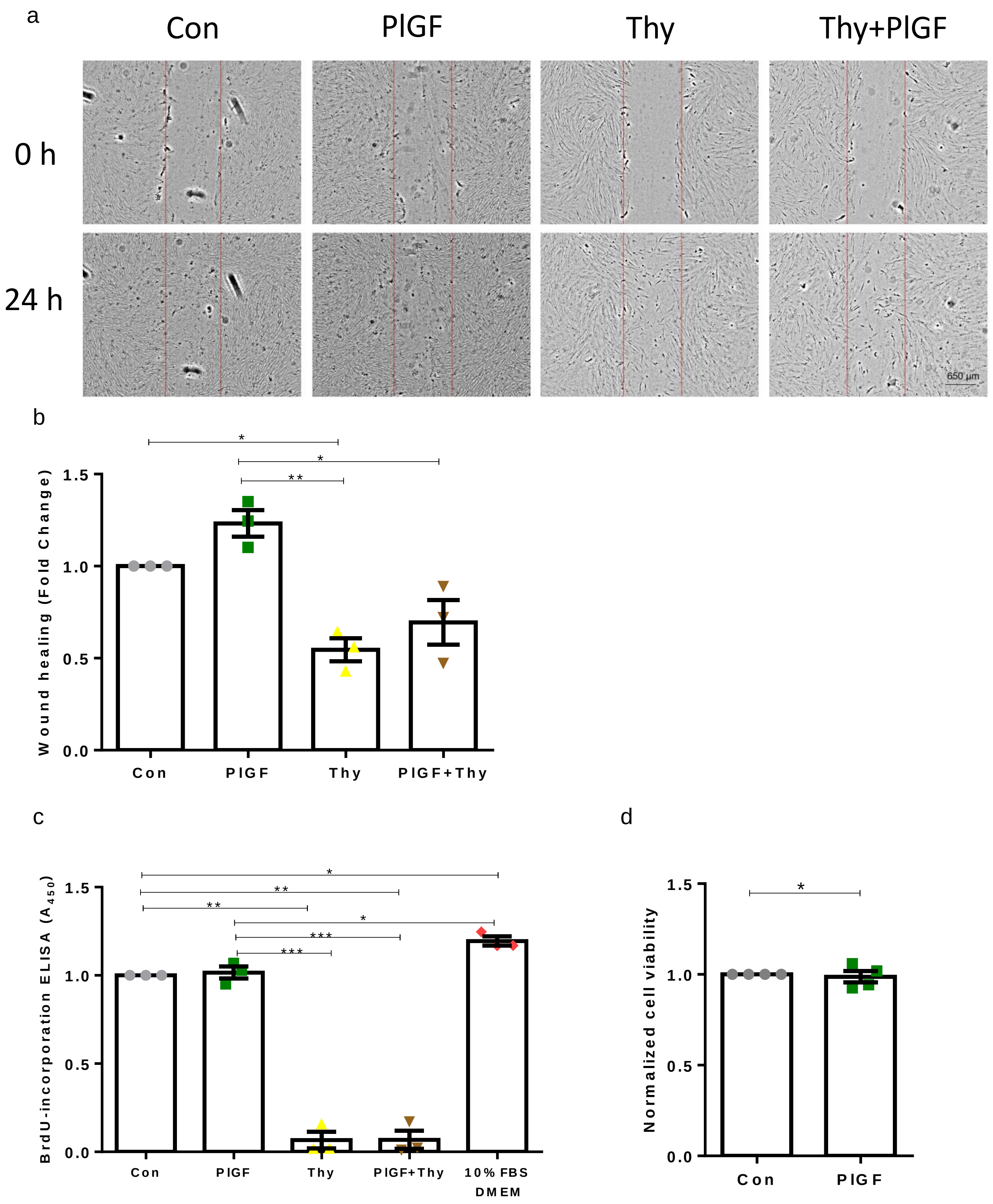

Fig S3 : a) Bright field images of wound healing scratch assay evaluated at 0h and 24 h. b) Arithmetic mean ± SEM of wound healing rate on EnSCs treated with PlGF ± thymidine (thy). c) Arithmetic mean ± SEM of BrdU cell proliferation assay on EnSCs treated with PlGF ± thymidine (thy). d) Arithmetic mean ± SEM of MTS cell viability assay on EnSCs treated with PlGF. All the above data represented here is normalized to control cells. An unpaired t test with Welch's correction was used to test for statistical significance

Fig S4

a

Con_EnSCs

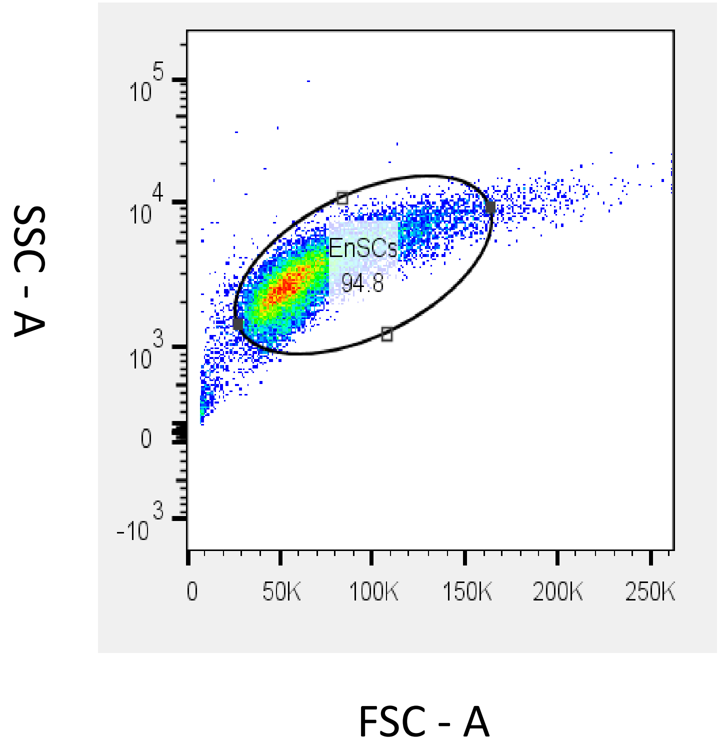

b

PlGF_EnSCs

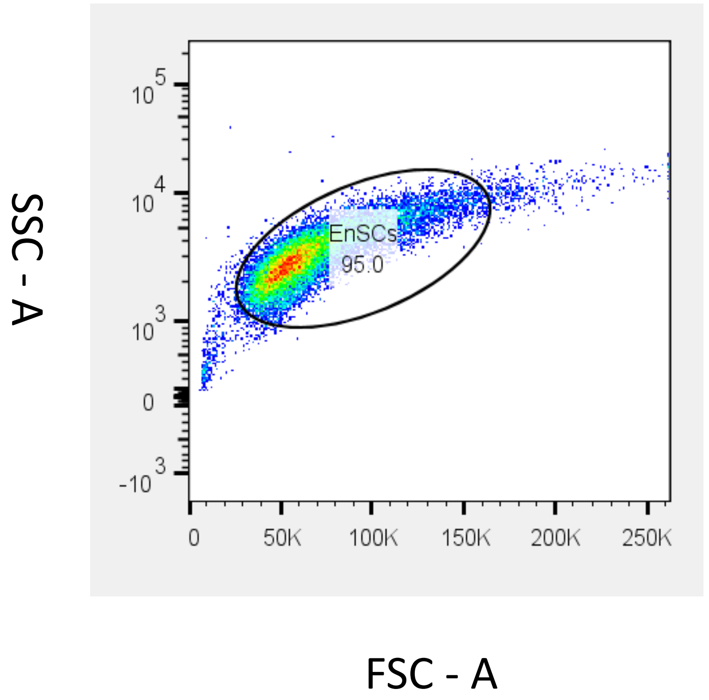

Fig S4: Gating strategy for single cells, illustrated in human endometrial stromal cell (EnSCs) population: Forward (FSC-A) and side scatter (SSC-A) are adjusted to minimize events on the axes, resulting in a single-cell population including > 80 % of total cells (a- Con, b- PlGF). Each dot or point on the plot represents an individual cell that has passed through the laser. Gating strategy has been applied on EnSCs population to exclude debris, dead cells and doublets. Cells gated on FSC-A versus SSC-A result in histograms specific to fluorophores (DNase I and Phalloidin) as depicted in both Figure 3a and 6a.

Fig S5

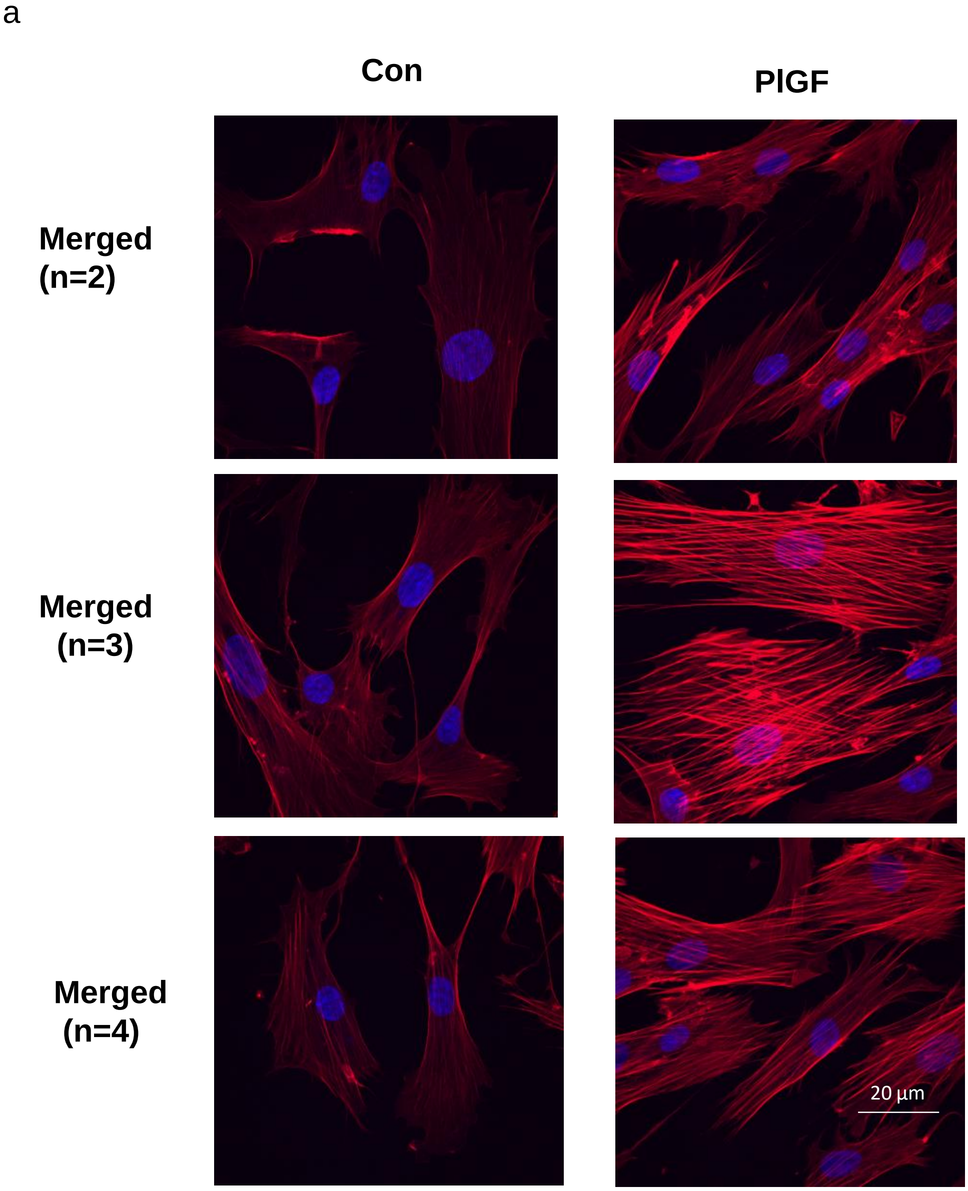

Fig S5: a. Representative immunofluoroscence images of EnSCs stained for phalloidin (F actin-red) and DAPI (nucleus – blue).

Fig S6

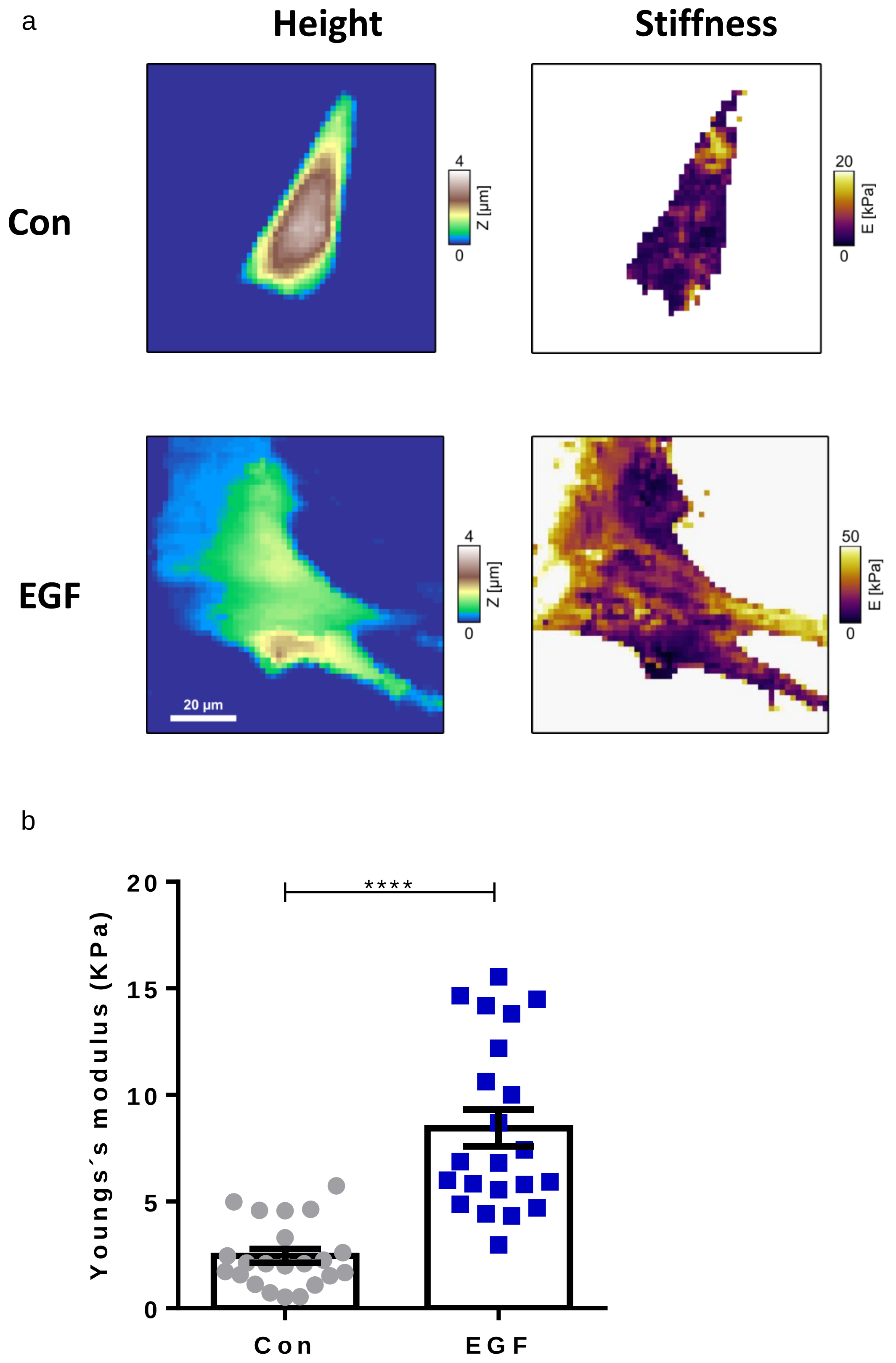

Fig S6 : a. AFM analysis of cell stiffness and cell morphology in EnSCs treated with EGF (100 ng/ml) for 24 hours. Representative height images and AFM stiffness images measuring the Young's modulus. (b) Arithmetic mean ± SEM of Young's modulus (cell stiffness. Non-parametric Mann-Whitney analysis was used to test for statistical significance.

Fig S7

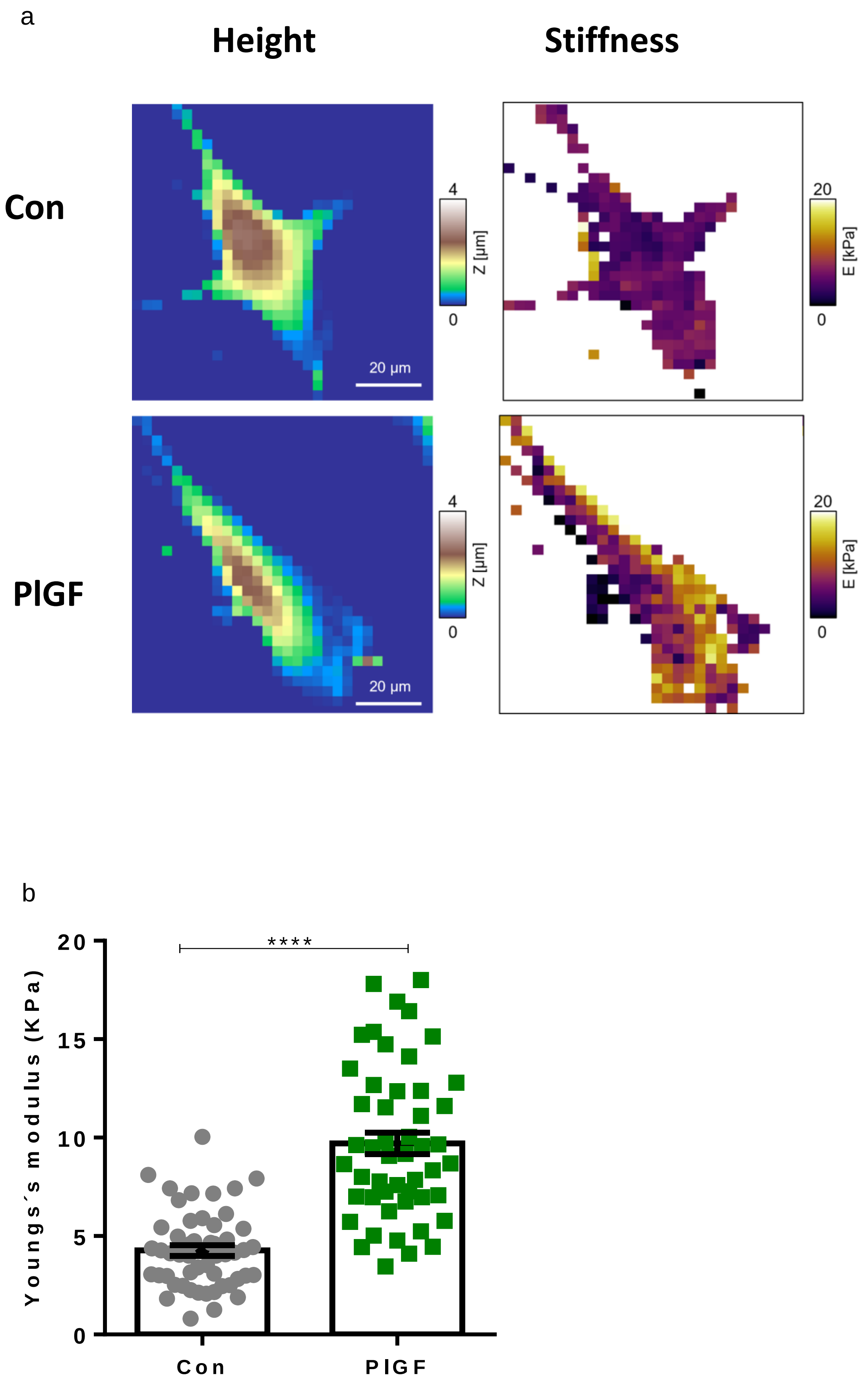

Fig S7 : a. AFM analysis of cell stiffness and cell morphology of EnSCs grown on PDMS coated petridishes. EnSCs were treated with PlGF (20 ng/ml) for 6 days, Representative height images and AFM stiffness images measuring the Young's modulus. (b) Arithmetic mean ± SEM of Young's modulus (cell stiffness). Non-parametric Mann-Whitney analysis was used to test for statistical significance.

Fig S8

a

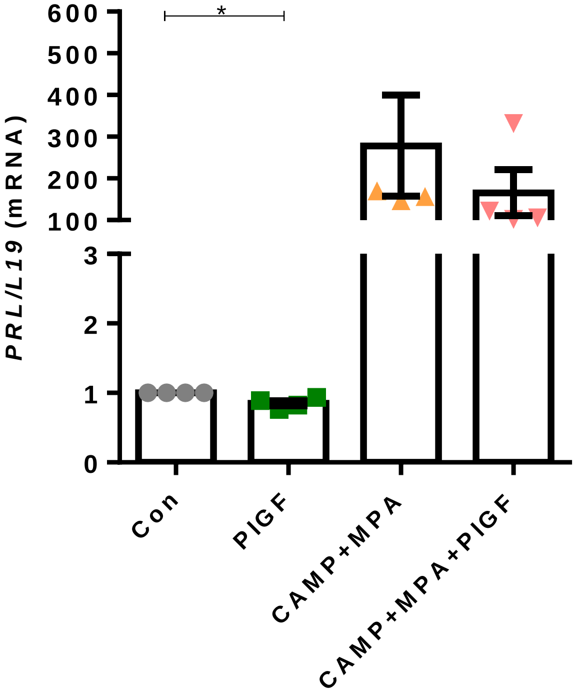

b

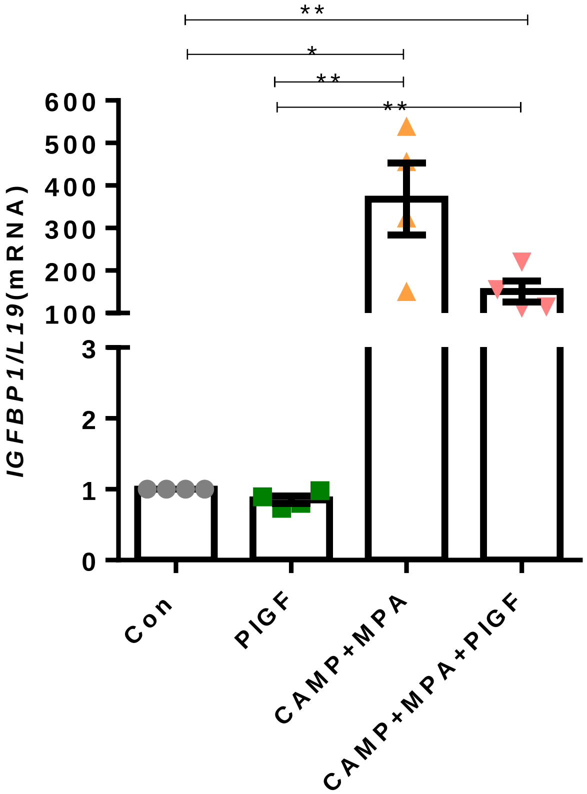

Fig S8: a) Arithmetic mean ± SEM of PRL transcript levels in EnSCs on treatment with PlGF±cAMP+MPA (n=4, *, $p<0.05$). (b) Arithmetic mean ± SEM of IGFBP1 transcript levels in EnSCs on treatment with PlGF±cAMP+MPA (n=4, *, $p<0.05$, **, $p<0.01$). All the above data represented here is normalized to control cells. An unpaired t test with Welch's correction was used to test for statistical significance.

Fig S9

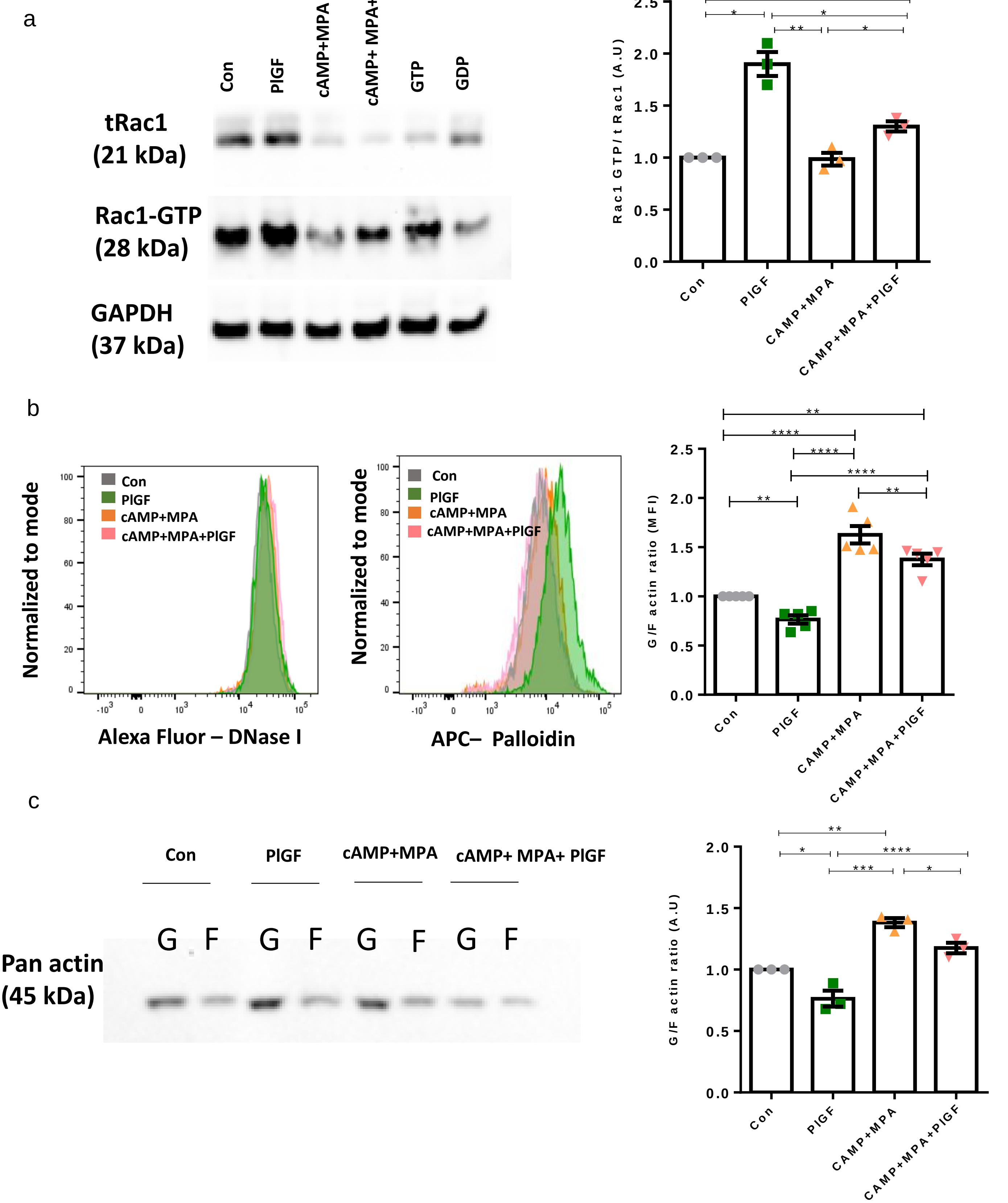

Fig S9 : a) Original WBs of tRac1,Rac1-GTP and arithmetic mean ± SEM of Rac1GTP levels / tRac1 ratio in EnSCs after 6 days treatment with PlGF ± cAMP+MPA. All the above data represented here is normalized to control cells. b) Representative original histogram of DNaseI (G-actin; Left) and Phalloidin (F-actin; Right) binding in EnSCs after 6 days treatment with PlGF ± cAMP+MPA and arithmetic mean ± SEM of G-actin over F-actin ratio in EnSCs after 6 days treatment with PlGF ± cAMP+MPA. c) Original western blots and arithmetic mean ± SEM of G-actin over F-actin ratio in EnSCs after 6 days treatment with PlGF ± cAMP+MPA. An unpaired t test with Welch's correction was used to test for statistical significance

Fig S10

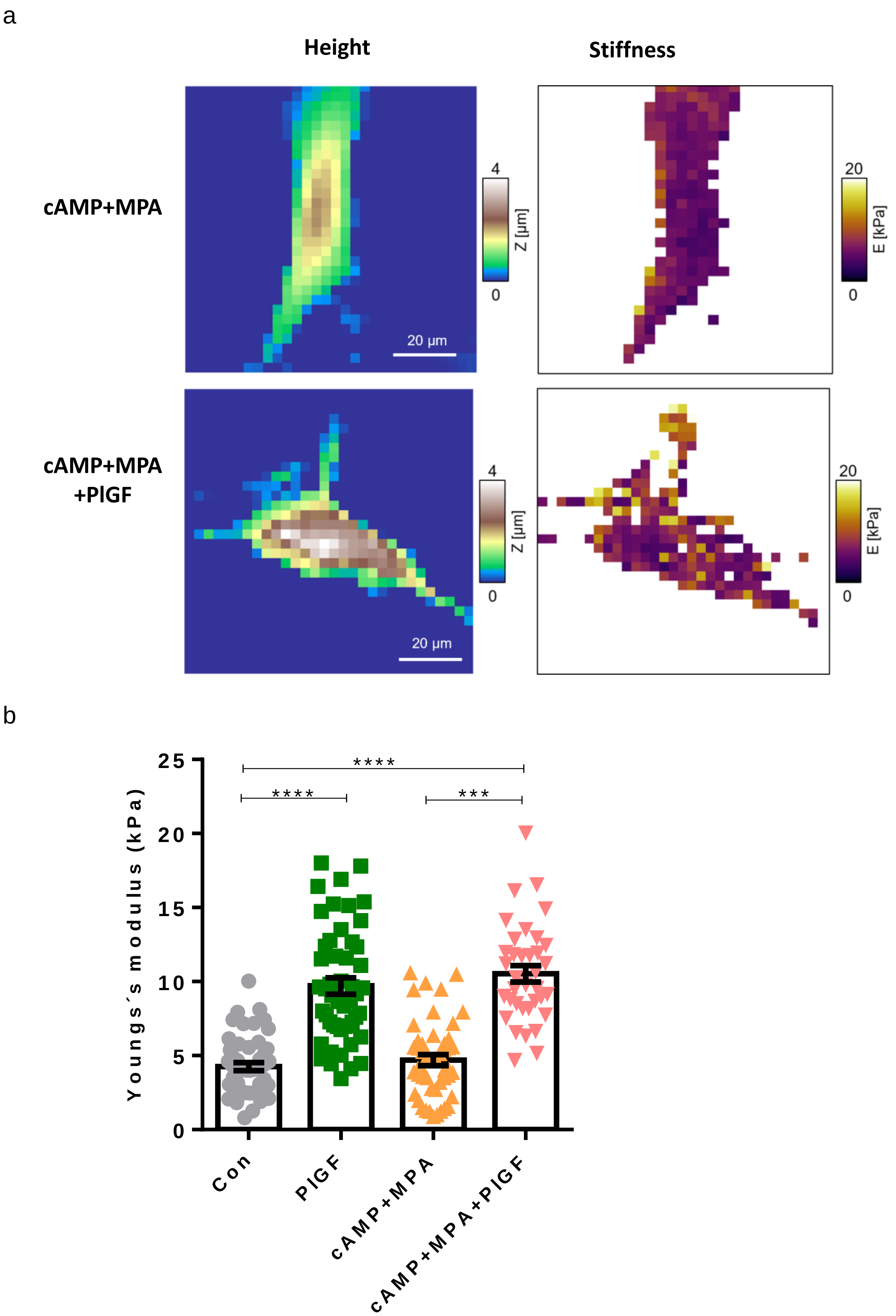

Fig S10: a. AFM analysis of cell stiffness and cell morphology of EnSCs grown on PDMS coated petridishes. EnSCs were treated with cAMP+MPA with or without PlGF for 6 days, Representative height images and AFM stiffness images measuring the Young's modulus. (b) Arithmetic mean ± SEM of Young's modulus (cell stiffness). Non-parametric Mann-Whitney analysis was used to test for statistical significance.

Fig S11

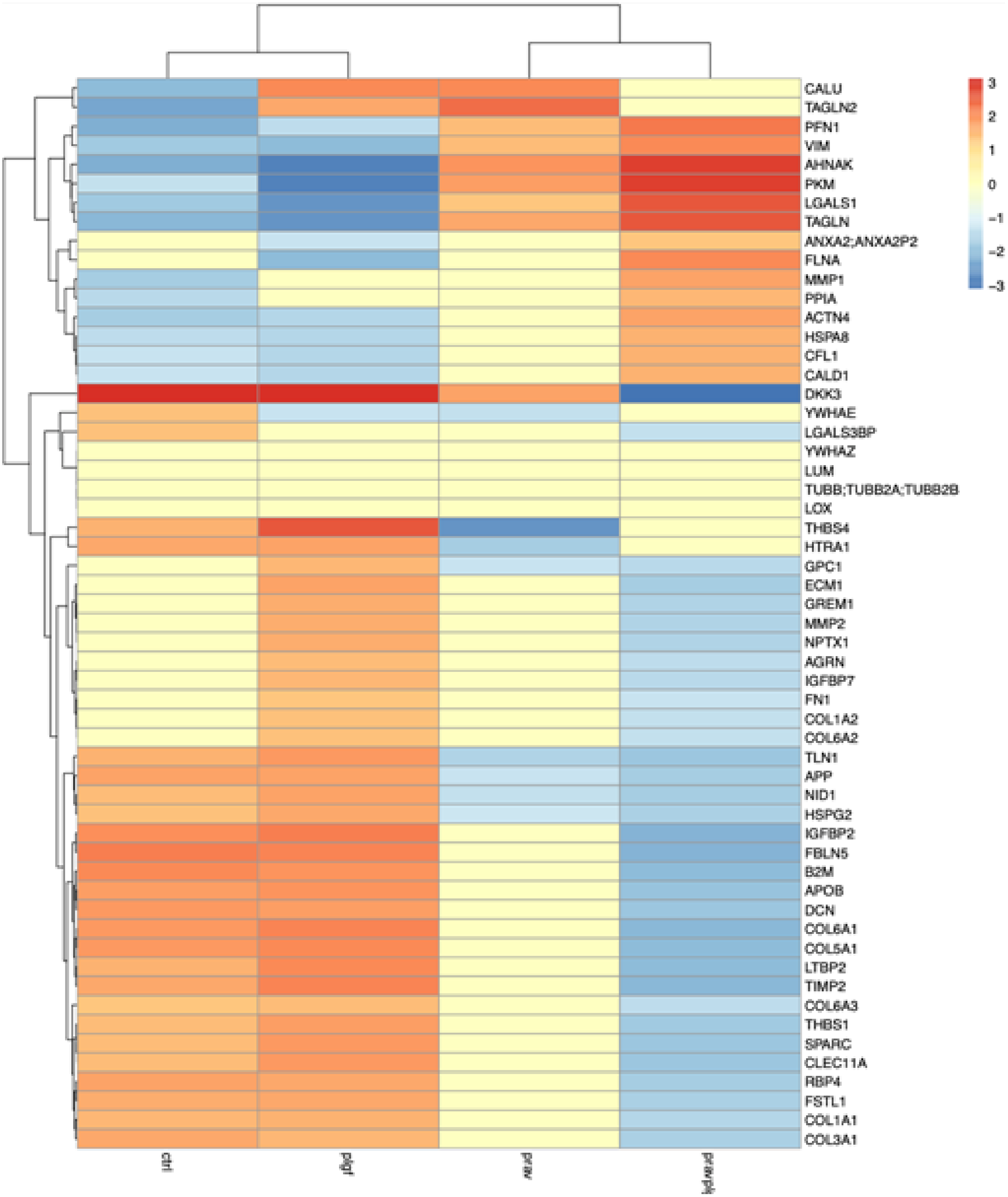

Fig S11: Heatmap showing the differentially regulated proteins expressed in different treatment group (Con/PlGF/Prav/PlGF+Prav) in EnSCs following global proteomic analysis.

Excessive endometrial PlGF- Rac1 signalling underlies endometrial cell stiffness linked to pre-eclampsia.

Supplementary Information

Original Western blots

Fig S12 Original western blot membranes of represented blots in figure 2c and 2h.

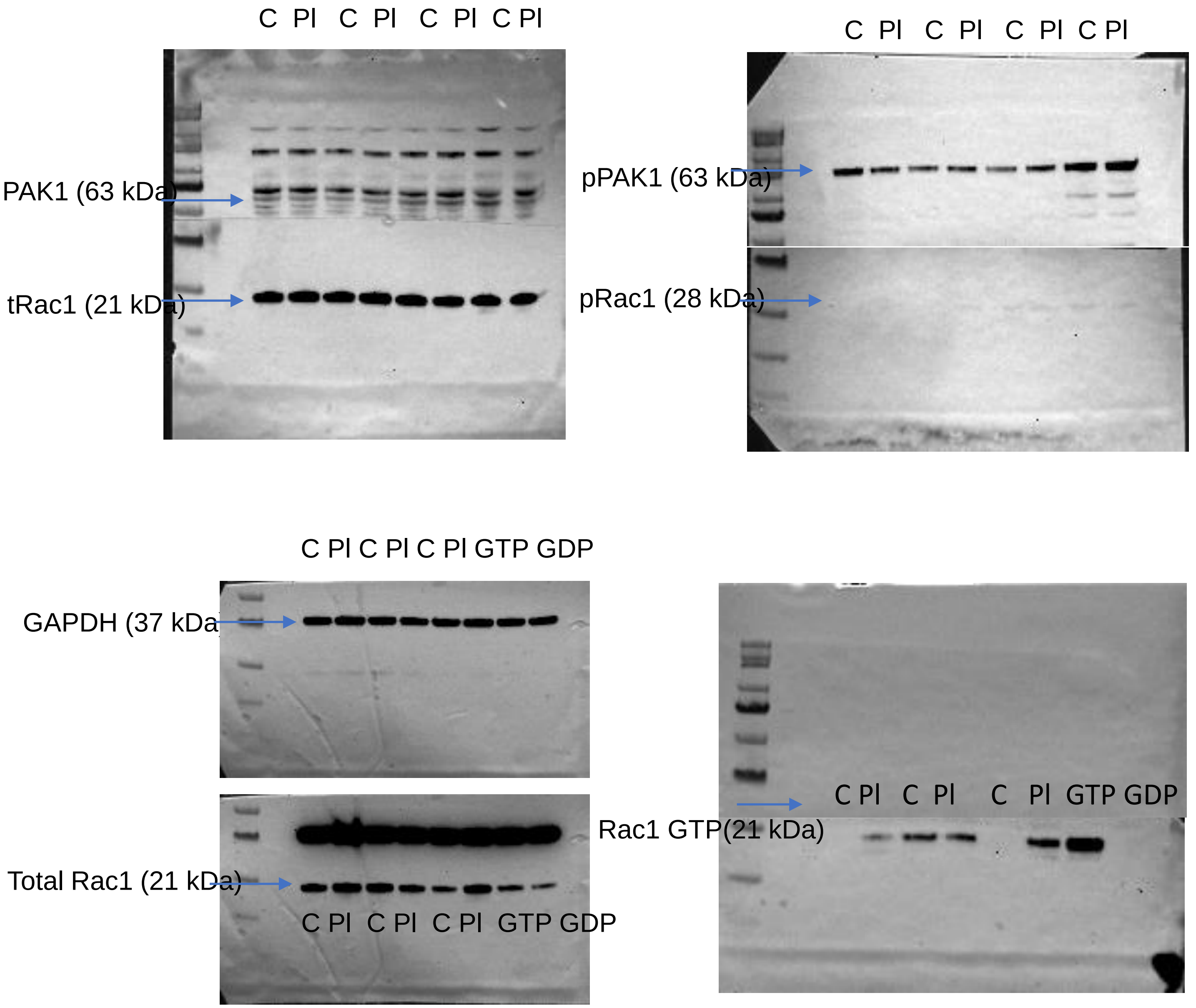

Original western blot membranes of represented blots in figure 3c

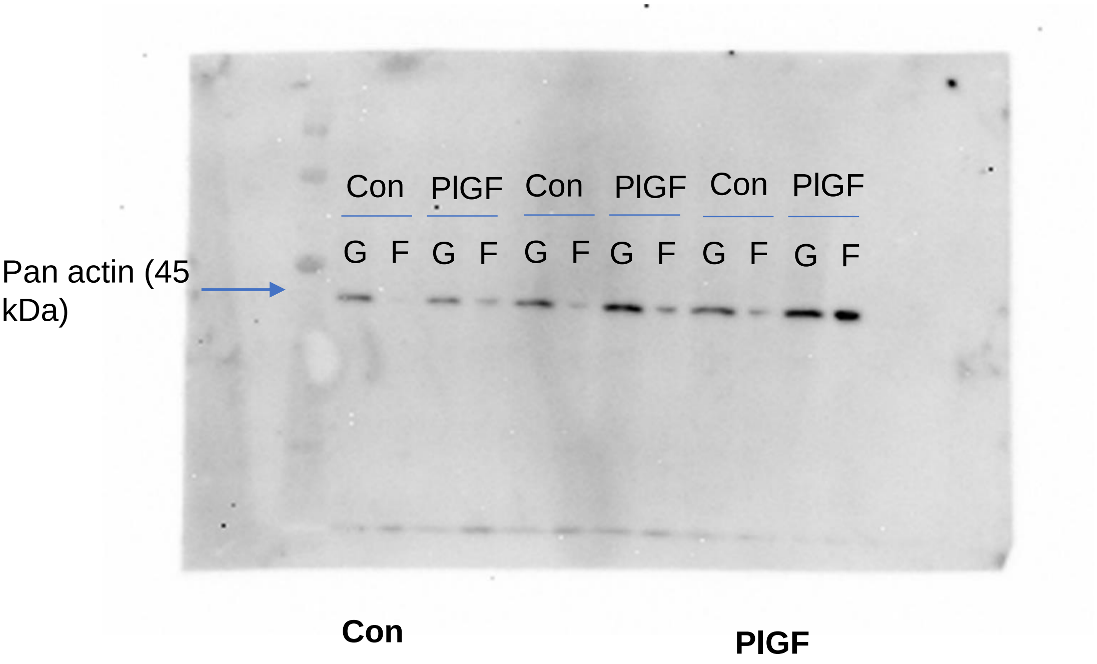

Original western blot membranes of the represented blots in figure 4a.

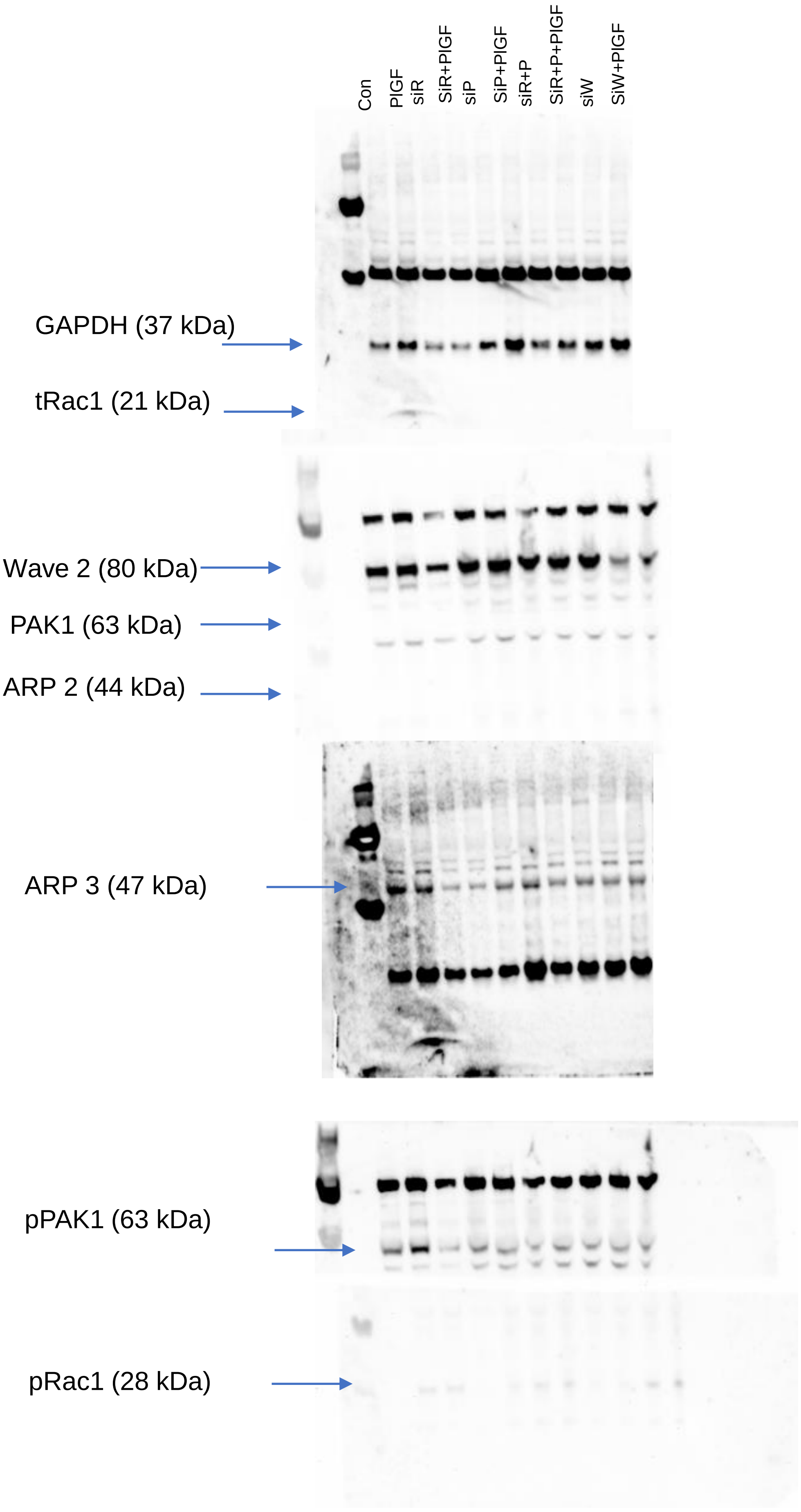

Original western blot membranes of represented blots in figure 5b.

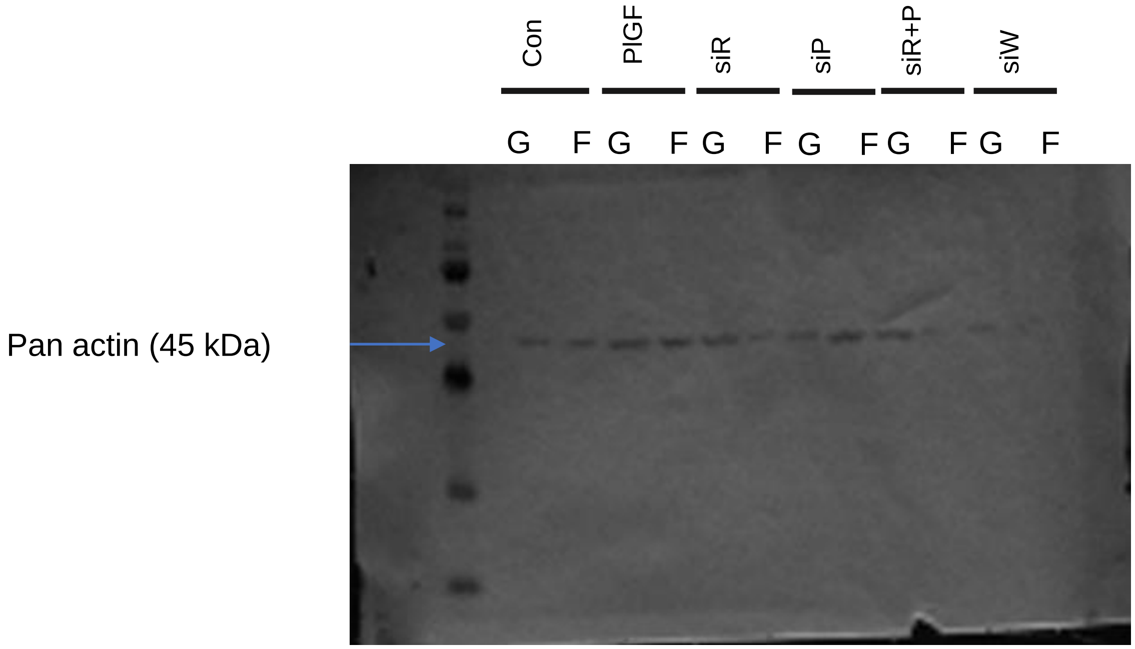

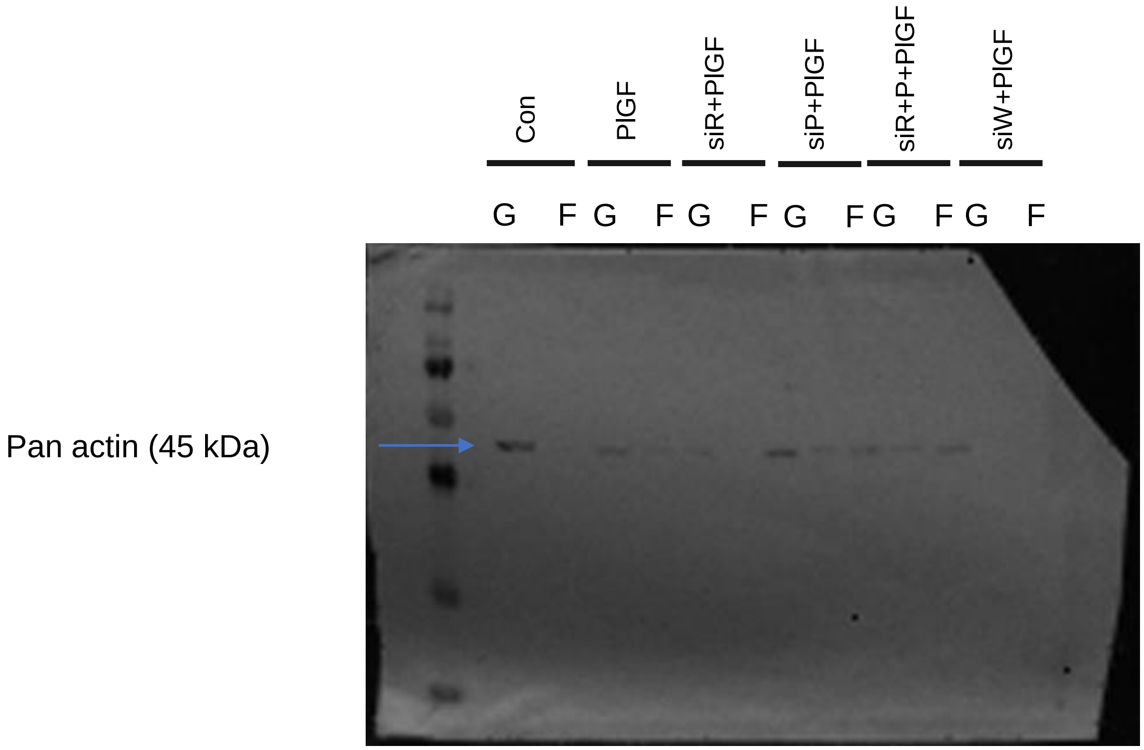

Original western blot membranes of represented blots in figure 6c and 6e.

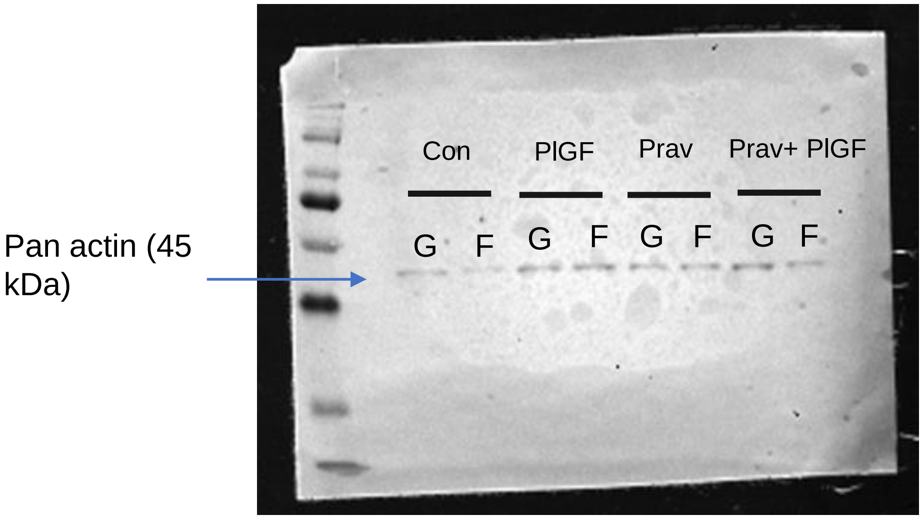

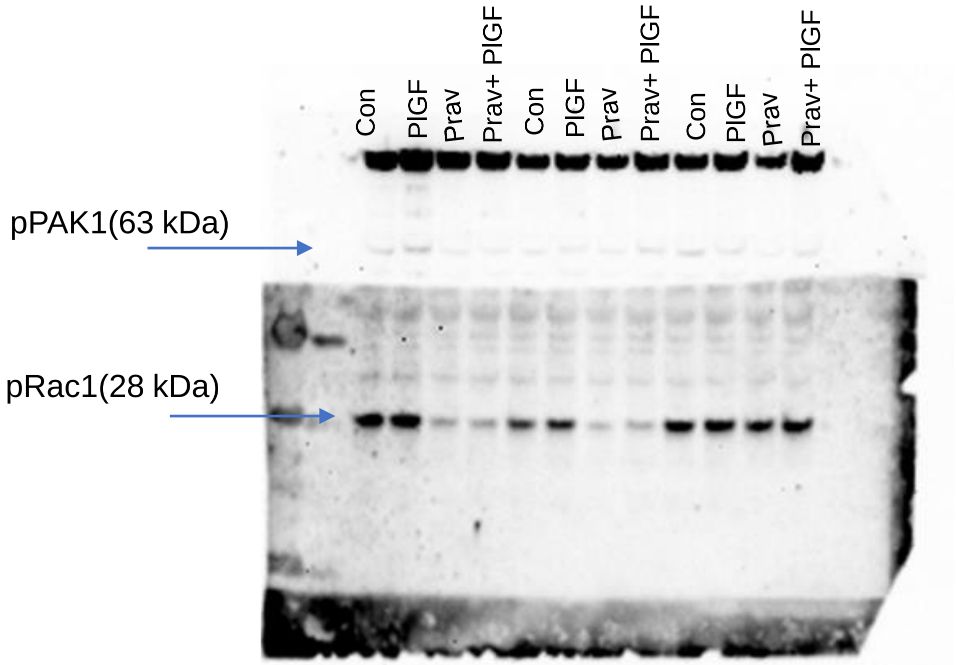

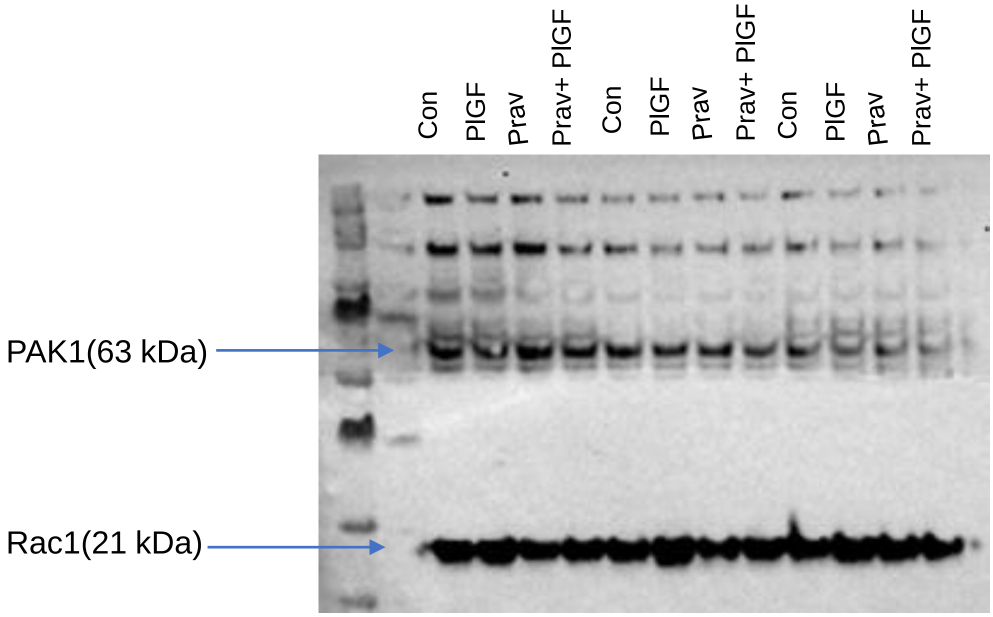

Supplement: Supplementary file 1 — Supplementary Information (new) [file 42003_2024_6220_MOESM1_ESM.pdf]
