# Supplementary material for: Excessive endometrial PlGF- Rac1 signalling underlies endometrial cell stiffness linked to pre-eclampsia
Source: Commun Biol. 2024 May 4;7:530. doi: 10.1038/s42003-024-06220-7 (PMC11069541; doi:10.1038/s42003-024-06220-7)
Supplement: Supplementary file 2 — Description of Additional Supplementary Files [file 42003_2024_6220_MOESM2_ESM.pdf]

Description of Additional Supplementary Files

File name: Supplementary Data

Description: Source data behind the graphs in the figures.
